# The WNT7A/WNT7B/GPR124/RECK signaling module plays an essential role in mammalian limb development

**DOI:** 10.1242/dev.200340

**Published:** 2022-05-12

**Authors:** Yanshu Wang, Arjun Venkatesh, Jiajia Xu, Mingxin Xu, John Williams, Philip M. Smallwood, Aaron James, Jeremy Nathans

**Affiliations:** 1 Department of Molecular Biology and Genetics, Johns Hopkins University School of Medicine, Baltimore, MD 21205, USA; 2 Howard Hughes Medical Institute, Johns Hopkins University School of Medicine, Baltimore, MD 21205, USA; 3 Department of Pathology, Johns Hopkins University School of Medicine, Baltimore, MD 21205, USA; 4 Department of Neuroscience, Johns Hopkins University School of Medicine, Baltimore, MD 21205, USA; 5 Department of Ophthalmology, Johns Hopkins University School of Medicine, Baltimore, MD 21205, USA

**Keywords:** Wnt signaling, Blood-brain barrier, Limb development, Mouse

## Abstract

In central nervous system vascular endothelial cells, signaling via the partially redundant ligands WNT7A and WNT7B requires two co-activator proteins, GPR124 and RECK. WNT7A and RECK have been shown previously to play a role in limb development, but the mechanism of RECK action in this context is unknown. The roles of WNT7B and GPR124 in limb development have not been investigated. Using combinations of conventional and/or conditional loss-of-function alleles for mouse *Wnt7a*, *Wnt7b*, *Gpr124* and *Reck*, including a *Reck* allele that codes for a protein that is specifically defective in WNT7A/WNT7B signaling, we show that reductions in ligand and/or co-activator function synergize to cause reduced and dysmorphic limb bone growth. Two additional limb phenotypes – loss of distal *Lmx1b* expression and ectopic growth of nail-like structures – occur with reduced *Wnt7a*/*Wnt7b* gene copy number and, respectively, with *Reck* mutations and with combined *Reck* and *Gpr124* mutations. A third limb phenotype – bleeding into a digit – occurs with the most severe combinations of *Wnt7a*/*Wnt7b*, *Reck* and *Gpr124* mutations. These data imply that the WNT7A/WNT7B-FRIZZLED-LRP5/LRP6-GPR124-RECK signaling system functions as an integral unit in limb development.

## Abstract (precis)

**Summary:** Genetic analyses in mice show that the WNT7A/WNT7B-FRIZZLED-LRP5/LRP6-GPR124-RECK signaling system, first defined in the context of CNS angiogenesis and barrier development, also functions as an integral unit in limb development.

## INTRODUCTION

The vertebrate limb has been one of the premier systems for studying how diverse extracellular signaling pathways interact to control the development of a morphologically complex structure (Zuniga, 2015). Among these pathways, WNT signaling plays multiple roles in limb development. In the developing mouse limb between embryonic day (E)9.5 and E15.5, 15/19 WNTs and 10/13 extracellular WNT antagonists are expressed in a wide variety of patterns (Witte et al., 2009). Gain- and loss-of-function genetic analyses implicate WNT signaling in a correspondingly wide variety of developmental processes, including proximal-distal growth, dorsal-ventral patterning, and the differentiation of cartilage, bone, muscle, and joints (Parr and McMahon, 1995; Kengaku et al., 1998; Barrow et al., 2003; Guo et al., 2004; Day et al., 2005; Hill et al., 2005; 2006; Zhu et al., 2012).

In mice, *Wnt7a* loss-of-function mutations ventralize the mesenchyme in the dorsal half of the distal limb, as judged by the anatomy of the joints and tendons (Parr and McMahon, 1995; Parr et al., 1998). Between E9.5 and E11.5, *Wnt7a* expression is restricted to the dorsal limb ectoderm; in the ventral ectoderm, it is repressed by the homeobox transcription factor ENGRAILED-1 (EN1) (Cygan et al., 1997). *En1* loss-of-function mutations relieve *Wnt7a* repression and dorsalize the ventral limb, as judged by the presence of hair follicles and the absence of eccrine glands on the ventral (palmar) skin and the presence of nails on both the upper and lower surfaces of each digit (Loomis et al., 1996; Cygan et al., 1997). The expression of *Wnt7b*, a close homologue of *Wnt7a*, is also confined to the ectoderm, but its expression is more widespread, with expression in both ventral and dorsal ectoderm (Summerhurst et al., 2008; Witte et al., 2009). A role for *Wnt7b* in limb development has not been previously reported.

*Wnt7a* and *Wnt7b* act in numerous developmental processes outside of the limb. *Wnt7a* is required for Müllerian duct regression, female fertility and maturation of cerebellar synapses (Miller and Sassoon, 1998; Parr and McMahon, 1998; Hall et al., 2000). *Wnt7b* is required for placental, pulmonary and pancreatic development, and for normal macrophage function (Parr et al., 2001; Shu et al., 2002; Lobov et al., 2005; Rajagopal et al., 2008; Yu et al., 2009; Lin et al., 2010; Afelik et al., 2015). *Wnt7a* and *Wnt7b* also function redundantly to promote central nervous system (CNS) angiogenesis and blood-brain barrier (BBB) development (Stenman et al., 2008; Daneman et al., 2009).

In CNS vascular endothelial cells (ECs), WNT7A and WNT7B signaling via the FRIZZLED receptor and LRP5/LRP6 coreceptor complex is greatly enhanced by two membrane proteins, GPR124 (also known as ADGRA2) and RECK (Zhou and Nathans, 2014; Posokhova et al., 2015; Vanhollebeke et al., 2015; Cho et al., 2017). In *Gpr124* knockout (KO) and *Reck* KO embryos, defects in CNS angiogenesis closely resemble the defects seen in embryos that are mutant for both *Wnt7a* and *Wnt7b* (Chandana et al., 2010; Kuhnert et al., 2010; Anderson et al., 2011; Cullen et al., 2011; Cho et al., 2017). In postnatal mice, eliminating *Gpr124* or *Reck* in ECs leads to a loss of blood-brain barrier integrity when β-catenin signaling via NORRIN (NDP; a non-WNT ligand) is also inactivated (Zhou and Nathans, 2014; Cho et al., 2017). GPR124 is a seven-pass transmembrane protein with a large multi-domain extracellular N-terminus (Hamann et al., 2015). RECK is a multidomain GPI-anchored plasma membrane protein that functions as both a WNT7A/WNT7B co-activator and a metalloprotease inhibitor (Takahashi et al., 1998; Oh et al., 2001). WNT7A/WNT7B activation requires the RECK amino-terminal CC-domains, whereas metalloproteinase inhibition is presumably mediated by the more C-terminal Kazal motif (Takahashi et al., 1998; Eubelen et al., 2018; Cho et al., 2019).

Although *Reck* KO mice die at E10.5, hypomorphic *Reck* mutants, in which deletion of exon 2 leads to reduced expression of an altered protein, survive to term and exhibit fore limb defects similar to the defects observed in *Wnt7a* KO mice (Yamamoto et al., 2012). This observation suggested the possibility that the WNT7A/WNT7B-FRIZZLED-LRP5/LRP6-GPR124-RECK signaling system, defined in the context of the CNS vasculature, might also function in the developing limb. To test this idea, we have studied the limb phenotypes produced by various combinations of *Wnt7a*, *Wnt7b*, *Gpr124* and *Reck* alleles. These experiments were modeled on an analogous series of gene dosage experiments in which progressively greater losses of β-catenin signaling components produced progressively more severe defects in CNS angiogenesis and BBB integrity (Zhou et al., 2014; Cho et al., 2017; 2019; Wang et al., 2018). The phenotypes and genetic interactions that we observe in the limb strongly support the idea that WNT7A, WNT7B, GPR124 and RECK function together in the limb, as they do in the brain.

## RESULTS

### *Wnt7a* and *Wnt7b* function additively in limb development

**Fig. 1.**
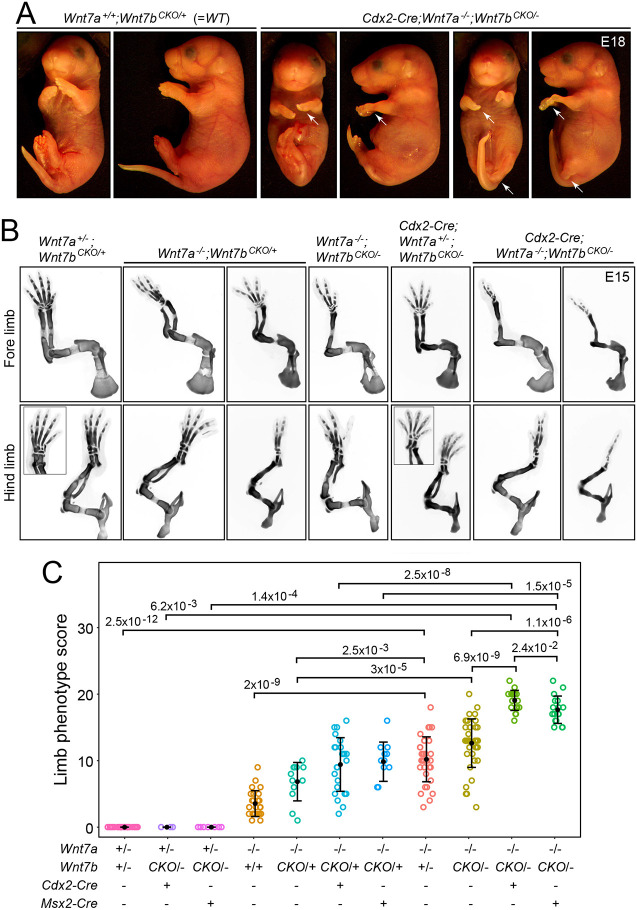
**Synergy between loss of *Wnt7a* and loss of *Wnt7b* in the severity of limb development phenotypes.** (A) Littermate WT (left) and *Cdx2-Cre;Wnt7a^−/−^;Wnt7b^CKO/−^* (right) E18 mice showing severe truncations of fore and hind limbs (white arrows) following efficient Cre-mediated recombination in the caudal embryo and less efficient Cre-mediated recombination in the rostral embryo. Front and side views of one WT and two mutant embryos. (B) Alcian Blue staining at E15 showing progressively more severe truncations with progressively reduced *Wnt7a* and *Wnt7b* function (from left to right). Insets, images of the foot rotated to more clearly show the digits. (C) Quantification of E15 limb skeletal defects with progressively reduced *Wnt7a* and *Wnt7b* function (from left-to-right). The scoring procedure is described in the Materials and Methods section. In C, a comparison between the fourth and fifth genotypes (*Wnt7a^−/−^;Wnt7b^+/+^* versus *Wnt7a^−/−^;Wnt7b^CKO/+^*) and between the eighth and ninth genotypes (*Wnt7a^−/−^;Wnt7b^+/−^* versus *Wnt7a^−/−^;Wnt7b^CKO/−^*) shows a modestly greater phenotype score with the *Wnt7b CKO* allele compared with the WT allele, suggesting that the introduction of *loxP* sites may be reducing *Wnt7b* expression. *P*-values were calculated using the non-parametric Mann–Whitney–Wilcoxon test. Error bars show the mean±s.d. Each circle represents a single limb.

As noted in the Introduction, a role for *Wnt7b* in limb development has not yet been reported. To explore this question, we examined the phenotypes of *Wnt7b* KO and conditional KO (CKO) alleles in wild-type (WT), *Wnt7a^+/−^* and *Wnt7a^−/−^* backgrounds. As *Wnt7b^−/−^* embryos die at mid-gestation from placental defects (Parr et al., 2001), we studied *Wnt7b^CKO/−^* and *Wnt7b^CKO/+^* combinations in which the *Wnt7b^CKO^* allele was inactivated by *Cdx2-Cre*, which is expressed in the early embryo but not the placenta and with a caudal>rostral pattern (Hinoi et al., 2007; Chang et al., 2016; Suh et al., 2019), and by *Msx2-Cre*, which is expressed in limb ectoderm (Sun et al., 2000) (Fig. 1). [More specifically, the *Msx2-Cre* transgene is expressed in the fore limb apical ectodermal ridge (AER) starting at ∼E10 and then its expression domain expands to include the ventral ectoderm of the fore limb by ∼E10.5; in the hind limb, the *Msx2-Cre* transgene is expressed broadly in the ectoderm, including the AER starting at ∼E9.5 (Sun et al., 2000).] In this, and most of the genetic interaction analyses described below, the core approach was to examine Alcian Blue-stained and benzyl benzoate:benzyl alcohol (BBBA)-clarified skeletons at E15. Skeletal phenotypes were quantified with a scoring system in which one point was assigned for each missing digit and two points for a missing distal limb bone (ulna or fibula). These different scores reflect the greater severity of limb defects associated with loss of either the ulna or fibula. Specifically, digit loss was often seen without loss of the ulna or fibula, but loss of the ulna or fibula was only seen in limbs that were also missing one or more digits. The numbers of embryos that were quantified are listed in Table S1. Fig. S1, with images of entire embryos, shows that the bone development defects are confined to the limbs. Fig. S2 shows an example of the corresponding adult phenotypes for WT and *Wnt7a^−/−^;Wnt7b^+/−^* limbs, using micro-computed tomography (μCT).

*Wnt7a* is expressed in dorsal limb ectoderm starting at ∼E9.5; and *Wnt7b* is expressed in both dorsal and ventral limb ectoderm at ∼E10.5 with ventral>dorsal expression at ∼E11.5 (Parr and McMahon, 1995; Summerhurst et al., 2008; Witte et al., 2009). Fig. 1 shows that loss of *Wnt7b* function throughout the hind limb (*Cdx2-Cre;Wnt7b^CKO/−^*) or in limb ectoderm (*Msx2-Cre;Wnt7b^CKO/−^*, the site of *Wnt7a* and *Wnt7b* expression; Sun et al., 2000; Summerhurst et al., 2008; Witte et al., 2009) produced no visible phenotype in either *Wnt7a^+/+^* or *Wnt7a^+/−^* backgrounds (Fig. 1B,C). However, in a *Wnt7a^−/−^* background, which typically leads to loss of one digit per foot and, in some cases, loss of the ulna (front limb) and/or fibula (hind limb), the additional loss of one allele of *Wnt7b* – in the form of *Wnt7b^+/−^*, *Cdx2-Cre;Wnt7b^CKO/+^*, or *Msx2-Cre;Wnt7b^CKO/+^* – increased the severity of the skeletal defects, as assessed by loss of additional digits, the ulna and/or the fibula (Fig. 1B,C). The limb defects in a *Wnt7a^−/−^* background were even more severe when combined with loss of both *Wnt7b* alleles – in the form of *Cdx2-Cre;Wnt7b^CKO/−^* or *Msx2-Cre;Wnt7b^CKO/−^* – with many feet showing only one or two digits and uniform loss of the ulna and fibula (Fig. 1A-C). The most severe limb phenotypes occasionally featured localized bleeding into a digit, highlighted by vertical red arrows in Fig. S1. For the genotypes shown in Fig. 1C, which are presented from left to right in order of increasing gene loss, comparisons can be made between genotypes with *Wnt7a^+/−^* versus *Wnt7a^−/−^* (left three versus right eight), between genotypes with no *Cre* versus *Cdx2-Cre* versus *Msx2-Cre* (for each group of three adjacent genotypes), and between genotypes with *Wnt7b^+/−^* versus *Wnt7b^CKO/−^* and *Wnt7b^+/+^* versus *Wnt7b^CKO/+^*.

In sum, loss of *Wnt7b* alone or in combination with loss of one copy of *Wnt7a* produces no apparent defect, loss of *Wnt7a* alone produces a moderate defect, and loss of *Wnt7a* together with one or both copies of *Wnt7b* produces the most severe defects in limb skeletal development. These data indicate that *Wnt7a* and *Wnt7b* play partially redundant roles in limb skeletal development. The data further show that step-wise reductions in WNT7A/WNT7B signaling lead to step-wise increases in the severity of the limb phenotype. Finally, the close similarity in phenotypic severity produced by *Cdx2-Cre* and *Msx2-Cre* drivers indicates that the principal source of WNT7B is the ectodermal territory in which *Msx2-Cre* is active.

### Genetic interactions between *Wnt7a*, *Gpr124* and *Reck* in limb development

**Fig. 2.**
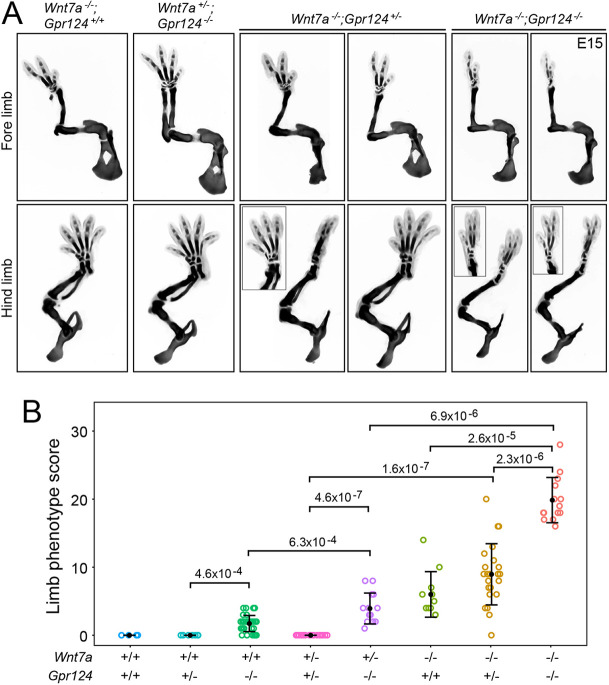
**Synergy between loss of *Gpr124* and loss of *Wnt7a* in the severity of limb development phenotypes.** (A) Alcian Blue staining of the skeleton at E15 showing progressively more severe truncations with progressively reduced *Wnt7a* and/or *Gpr124* function (from left to right). Insets, images of the foot rotated to more clearly show the digits. (B) Quantification of E15 limb skeletal defects with progressively reduced *Wnt7a* and/or *Gpr124* function (from left-to-right). The scoring procedure is described in the Materials and Methods section. *P*-values were calculated using the non-parametric Mann–Whitney–Wilcoxon test. Error bars show the mean±s.d. Each circle represents a single limb.

To explore the possibility that GPR124 might play a role in limb development, we characterized E15 limbs in embryos with heterozygous or homozygous loss of *Gpr124*, either alone or in combination with heterozygous or homozygous loss of *Wnt7a* (Fig. 2A,B). In Fig. 2B, the genotypes are presented in three groups in order of increasing gene loss from left to right: *Wnt7a^+/+^*, *Wnt7a^+/−^* and *Wnt7a^−/−^*, each in combination with different numbers of *Gpr124* alleles. *Gpr124^+/−^* embryos showed no skeletal defects, but *Gpr124^−/−^* embryos showed skeletal defects similar to, but milder than, those seen with loss of *Wnt7a*. Interestingly, although *Wnt7a^+/−^;Gpr124^+/−^* embryos showed no limb defects, *Wnt7a^+/−^;Gpr124^−/−^* embryos showed defects more severe than those of *Gpr124^−/−^* embryos. Similarly, although *Wnt7a^−/−^;Gpr124^+/−^* embryos showed phenotypes that were only marginally more severe than those of *Wnt7a^−/−^* embryos, *Wnt7a^−/−^;Gpr124^−/−^* embryos exhibited defects that were substantially more severe than those of *Wnt7a^−/−^;Gpr124^+/−^* embryos, with many feet showing only one or two digits and uniform loss of the ulna and fibula (Fig. 2A,B). These data are consistent with a role for GPR124 in co-activating WNT7A/7B signaling in the limb, analogous to its role in CNS ECs.

**Fig. 3.**
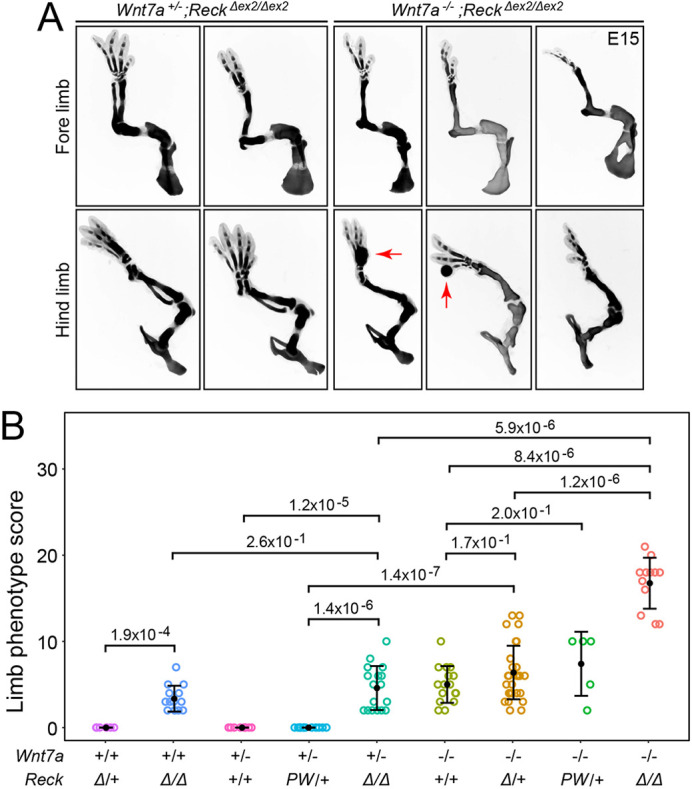
**Synergy between *Reck* and *Wnt7a* loss in the severity of limb development phenotypes.** (A) Alcian Blue staining of the skeleton at E15 showing that, in a *Reck^Δex2/Δex2^* background, reducing the number of WT copies of *Wnt7a* from one to zero increases the severity of fore and hind limb skeletal defects. Red arrows point to regions with local bleeding (see Fig. S1 for additional examples). (B) Quantification of E15 limb skeletal defects with progressively reduced *Wnt7a* and/or *Reck* function (from left to right). *Δ*, *Reck^Δex2^*; *PW*, *Reck^PW^*. The scoring procedure is described in the Materials and Methods section. *P*-values were calculated using the non-parametric Mann–Whitney–Wilcoxon test. Error bars show the mean±s.d. Each circle represents a single limb.

Homozygosity for a hypomorphic *Reck* allele (a deletion of *Reck* exon 2 that greatly reduces RECK abundance; Cho et al., 2019; abbreviated here as *Reck^Δex2^*) results in limb defects comparable in severity with those caused by loss of *Wnt7a* (Yamamoto et al., 2012). Fig. 3 shows the effects of combinations of *Wnt7a* and *Reck* loss-of-function. In Fig. 3B, the genotypes are presented in three groups in order of increasing gene loss from left to right: *Wnt7a^+/+^*, *Wnt7a^+/−^* and *Wnt7a^−/−^*, each in combination with different numbers or types of *Reck* alleles. In comparing *Wnt7a^+/+^;Reck^Δex2/Δex2^* versus *Wnt7a^+/−^;Reck^Δex2/Δex2^* and *Wnt7a^−/−^;Reck^+/+^* versus *Wnt7a^−/−^*;*Reck^Δex2/+^* limb phenotypes, we observed small (and statistically non-significant) enhancements in the skeletal phenotype with the additional loss of a WT *Wnt7a* or *Reck* allele (Fig. 3A,B). In contrast, there is a large enhancement in the limb phenotype in comparing *Wnt7a^−/−^;Reck^Δex2/+^* versus *Wnt7a^−/−^;Reck^Δex2/Δex2^* embryos (Fig. 3A,B). A CRISPR-generated *Reck* allele in which proline(P)^256^ and tryptophan(W)^261^ in the CC4 domain are substituted by alanine (*Reck^PW^*) disrupts the ability of RECK to stimulate WNT7A/7B signaling, but it has no effect on RECK abundance and cell surface localization, and it presumably has no effect on the metalloproteinase inhibitor activity (Cho et al., 2019). Therefore, we infer that any phenotypes caused by the *Reck^PW^* allele arise from a decrease in WNT7A/WNT7B signaling. In these experiments, the *Reck^PW^* allele behaved similarly to the *Reck^Δex2^* allele, showing no limb defects in the heterozygous state (*Reck^PW/+^*) and having little effect on the limb phenotype when combined in the heterozygous state with *Wnt7a^−/−^* (*Wnt7a^−/−^;Reck^+/+^* versus *Wnt7a^−/−^;Reck^PW/+^*) (Fig. 3B). As *Reck^PW/PW^* embryos do not survive to E15, their limb phenotype is unknown.

**Fig. 4.**
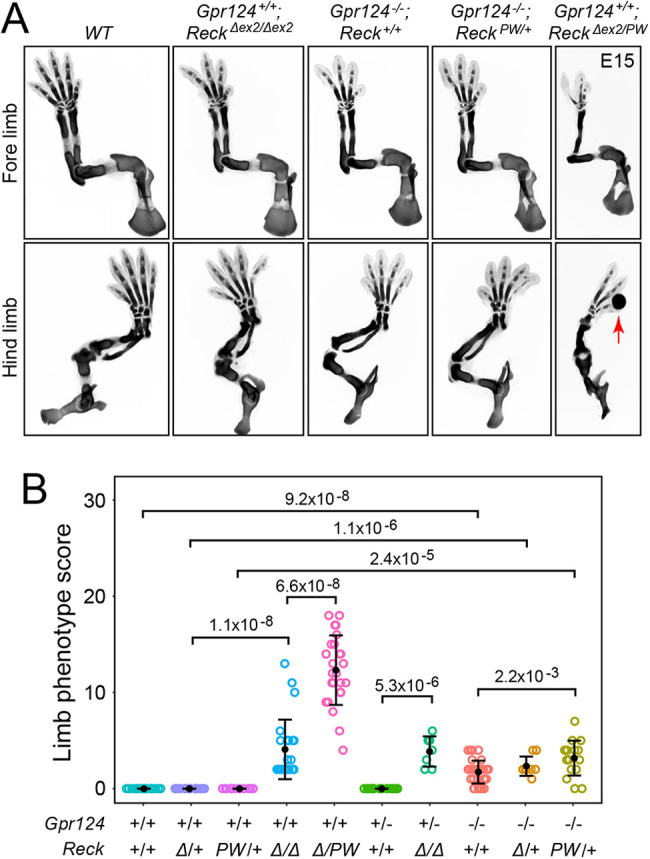
**Requirement for *Gpr124* and *Reck* function in limb development.** (A) Alcian Blue staining of the skeleton at E15 showing progressively more severe truncations with progressively reduced *Gpr124* and/or *Reck* function (from left to right). Red arrow points to a region with local bleeding (see Fig. S1 for additional examples). (B) Quantification of E15 limb skeletal defects with progressively reduced *Wnt7a* and/or *Gpr124* function. *Δ*, *Reck^Δex2^*; *PW*, *Reck^PW^*. The scoring procedure is described in the Materials and Methods section. *P*-values were calculated with the non-parametric Mann–Whitney–Wilcoxon test. Error bars show the mean±s.d. Each circle represents a single limb.

To further explore the roles of *Gpr124* and *Reck* and their potential synergy, embryos with various combinations of *Gpr124* and *Reck* loss-of-function alleles were analyzed (Fig. 4). The five genotypes shown at the left of Fig. 4B show this analysis for various combinations of *Reck* alleles, in order of increasing phenotypic severity from left to right. In the brain, the *Reck^Δex2/PW^* compound heterozygote shows a severe disruption of angiogenesis in the cortex and ganglionic eminences, suggesting that the combination of a RECK protein that is defective in WNT7A/WNT7B stimulation (encoded by the *Reck^PW^* allele) and an internally deleted RECK protein that accumulates to levels many fold lower than WT RECK (encoded by the *Reck^Δex2^* allele) is insufficient to support WNT7A/WNT7B signaling in CNS ECs. Similarly, the limbs in *Reck^Δex2/PW^* embryos have skeletal defects substantially more severe than those associated with *Reck^Δex2/Δex2^* (Fig. 4A,B), comparable with the phenotype seen in *Wnt7a^−/−^;Wnt7b^+/−^* limbs (compare Figs 1C and 4B). These data imply that RECK action in limb development is largely referable to its role as a WNT7A/WNT7B co-activator, which is eliminated in the *Reck^PW^* allele, and that the role of RECK as a metalloproteinase inhibitor is of secondary significance in this context.

If GPR124 and RECK cooperate in promoting WNT7A/WNT7B signaling in the limbs, as they do in CNS ECs, we would predict that simultaneously reducing the dosage of both genes might enhance the limb phenotype relative to the phenotypes seen with reductions in individual gene dosages. An initial set of crosses with conventional alleles showed minimal genetic interactions, with no measurable difference in phenotypic severity in comparing *Gpr124^+/+^;Reck^Δex2/Δex2^* versus *Gpr124^+/−^;Reck^Δex2/Δex2^* (fourth and seventh genotypes in Fig. 4B) and only a small increase in phenotypic severity in comparing *Gpr124^−/−^* versus *Gpr124^−/−^;Reck^Δex2/+^* or *Gpr124^−/−^;Reck^PW/+^* (rightmost three genotypes in Fig. 4B).

**Fig. 5.**
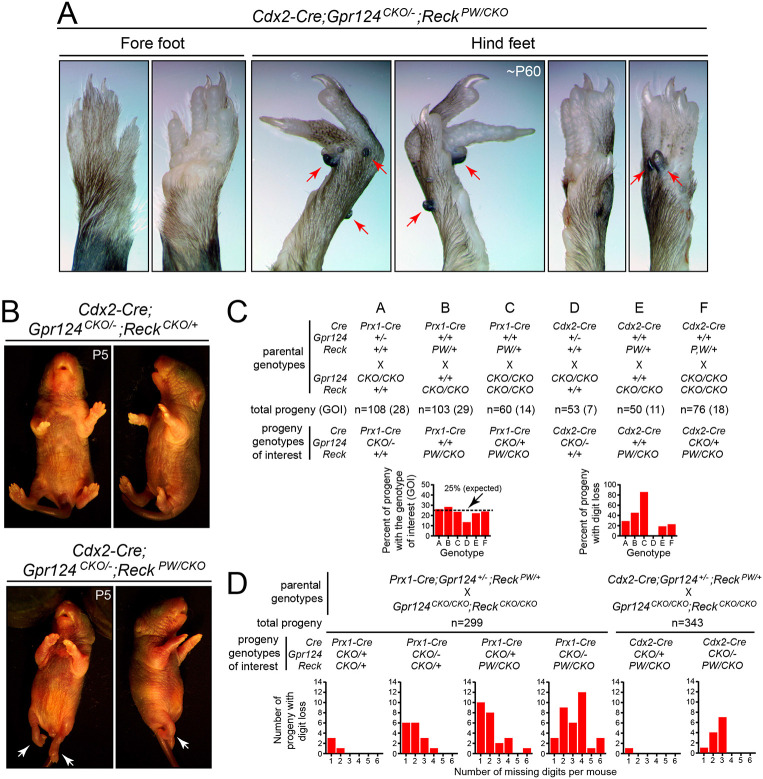
**Increased severity of limb defects with *Prx1-Cre*- and *Cdx2-Cre*-mediated loss of greater numbers of *Gpr124* and/or *Reck* alleles.** (A) In *Cdx2-Cre;Gpr124^CKO/−^;Reck^PW/CKO^* mice, adult fore feet (left) are essentially unaffected whereas hind feet (right) show digit loss, aberrant structure and ectopic nail-like structures (red arrows). Two views each of one fore foot and two hind feet. (B) A comparison between *Cdx2-Cre;Gpr124^CKO/−^;Reck^CKO/+^* and *Cdx2-Cre;Gpr124^CKO/−^;Reck^PW/CKO^* mice at P5 shows the severe hind limb defects caused by replacement of the one remaining WT *Reck* allele by *Reck^PW^*, which is defective specifically in WNT signaling (white arrows). (C,D) Genetic crosses to remove *Gpr124* and/or *Reck* in the caudal region of the early embryo (*Cdx2-Cre*) or in the limb mesenchyme (*Prx1-Cre*). The panels show the number of genotype-of-interest (GOI) progeny obtained relative to the number expected, the percent of progeny with loss of one or more digits (C), and the number of progeny with different numbers of missing digits (D). Parent and progeny genotypes for six crosses (labeled A-F) are shown in C and for two crosses are shown in D. Progeny were scored between P3 and P8.

A more extensive series of crosses with CKO alleles in combination with *Cdx2-Cre* or *Prx1-Cre* (a limb mesoderm-specific *Cre* line; Logan et al., 2002) permitted postnatal analyses of a larger number of progeny (>1000; Fig. 5). Consistent with the caudal>rostral pattern of *Cdx2-Cre* expression, mice with inactivation of *Gpr124^CKO^* and/or *Reck^CKO^* mediated by *Cdx2-Cre* showed defects in hind limbs but not fore limbs (Fig. 5A,B). In six crosses designed to inactivate as many as three of the four *Gpr124* and *Reck* alleles with each of the two *Cre* transgenes, postnatal progeny with the most extreme genotype appeared at the expected ∼25% frequency in five crosses and at modestly reduced frequency (13%) in one cross (Fig. 5C). In the allelic series with recombination mediated by *Prx1-Cre*, digit loss was observed in 29% of *Prx1-Cre;Gpr124^CKO/−^* progeny, 45% of *Prx1-Cre;Reck^PW/CKO^* progeny and 86% of *Prx1-Cre;Gpr124^CKO/+^;Reck^PW/CKO^* progeny, the latter having three of four *Gpr124* and *Reck* alleles mutated (Fig. 5C). In the allelic series with *Cdx2-Cre*-mediated recombination, digit loss was observed in 0% of *Cdx2-Cre;Gpr124^CKO/−^* progeny, 18% of *Cdx2-Cre;Reck^PW/CKO^* progeny, and 22% of *Cdx2-Cre;Gpr124^CKO/+^;Reck^PW/CKO^* progeny, the latter having three of four *Gpr124* and *Reck* alleles mutated (Fig. 5C).

In a cross targeting all four *Gpr124* and *Reck* alleles with *Prx1-Cre*, a clear trend was observed in which progressively more digits were lost with the inactivation of more alleles, with a rank order of phenotypic severity *Prx1-Cre;Gpr124^CKO/+^;Reck^CKO/+^*<*Prx1-Cre;Gpr124^CKO/−^;Reck^CKO/+^*<*Prx1-Cre;Gpr124^CKO/+^;Reck^PW/CKO^*<*Prx1-Cre;Gpr124^CKO/−^;Reck^PW/CKO^* (Fig. 5D). The analogous cross with *Cdx2-cre* produced milder phenotypes, with significant digit losses observed only with inactivation of all four alleles (Fig. 5A,D). These data are consistent with: a greater requirement for *Reck* than *Gpr124* in limb development; a moderate degree of genetic synergy/additivity between *Reck* and *Gpr124*; and milder defects produced by the *Reck* and *Gpr124* CKO alleles compared with the KO alleles, perhaps due to delayed timing or incompleteness of Cre-mediated recombination.

### Synergy between *Wnt7a* and *Tbx3* in limb development

TBX3 is a DNA-binding transcription factor and one of 17 T-box family members in mammals. TBX3 plays important roles in the development of the limbs, lungs, kidneys, mammary gland, inner ear and cardiac conduction system (Davenport et al., 2003; Emechebe et al., 2016; Lüdtke et al., 2016; Aydoğdu et al., 2018; Mohan et al., 2020; Kaiser et al., 2021). Analyses of *Tbx3* KO and CKO phenotypes in mice and heterozygous *TBX3* mutations in humans (the cause of ulnar-mammary syndrome; Khan et al., 2020), imply distinct roles for TBX3 in different limb compartments and at different stages of limb development. In mice, early inactivation of *Tbx3* causes defects in limb initiation, and later inactivation of *Tbx3* causes either syndactyly or polydactyly, depending on which compartment is affected (Emechebe et al., 2016). Of relevance to the present study, TBX3 functions as a component of the β-catenin-LEF/TCF-BCL9 transcriptional regulatory complex, as determined by ChIP-seq experiments with E10.5 mouse limb tissue and by reporter gene responses to TBX3 in HEK/293 cells carrying WT or CRISPR-inactivated components of this complex (Zimmerli et al., 2020).

**Fig. 6.**
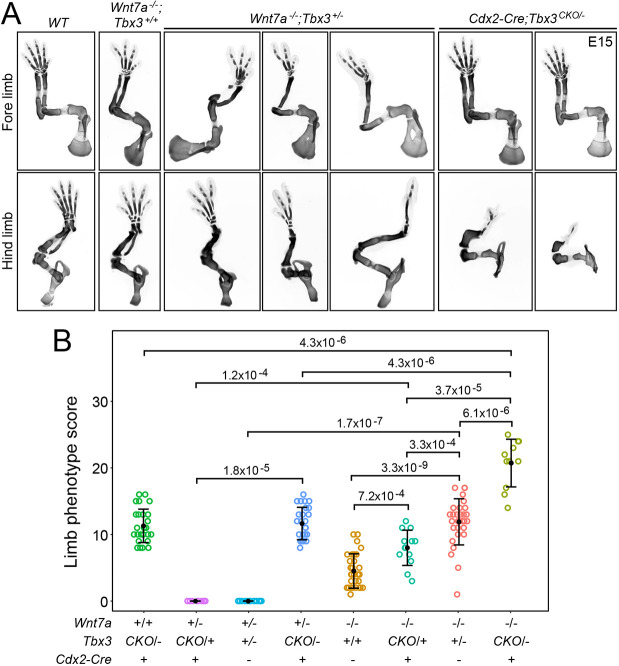
**Synergy between loss of *Tbx3* and loss of *Wnt7a* in the severity of limb development phenotypes.** (A) Alcian Blue staining of the skeleton at E15 showing progressively more severe truncations with progressively reduced *Tbx3* in a *Wnt7a^−/−^* background or with early embryonic loss of *Tbx3*. (B) Quantification of E15 limb skeletal defects with progressively reduced *Wnt7a* and/or *Tbx3* function. The scoring procedure is described in the Materials and Methods section. *P*-values were calculated with the non-parametric Mann–Whitney–Wilcoxon test. Error bars show the mean±s.d. Each circle represents a single limb.

To complement the biochemical and genomic analyses of TBX3 and the β-catenin transcriptional regulatory complex (Zimmerli et al., 2020), we asked whether *Wnt7a* and *Tbx3* exhibit genetic interactions in the limb. Although *Tbx3^+/−^* embryos have normal limbs, combining *Tbx3^+/−^* with *Wnt7a^−/−^* greatly increases the severity of the *Wnt7a^−/−^* limb phenotype, with loss of the ulna and fibula and loss of one or more digits per foot (Fig. 6A; compare the third, fifth and seventh genotypes in Fig. 6B). To assess the more severe phenotypes associated with complete or nearly complete inactivation of *Tbx3* in combination with inactivation of *Wnt7a*, while also circumventing the untoward effects of the *Tbx3^−/−^* genotype on embryonic development (including lethality at ∼E16; Davenport et al., 2003), we used *Cdx2-Cre*-mediated inactivation of a *Tbx3^CKO^* allele. This cross revealed an increased severity of the limb skeletal phenotype in *Cdx2-Cre;Tbx3^CKO/−^;Wnt7a^−/−^* embryos compared with *Cdx2-Cre;Tbx3^CKO/+^;Wnt7a^−/−^* or *Cdx2-Cre;Tbx3^CKO/−^;Wnt7a^+/−^* embryos (Fig. 6B, compare the eighth genotype with the sixth and fourth genotypes). Taken together, these *in vivo* analyses support a model in which *Wnt7a* and *Tbx3* act in the same pathway. They are, therefore, consistent with the model of Zimmerli et al. (2020) in which TBX3 participates directly in the transcriptional response to β-catenin signaling in the developing limb.

### Ectopic nail-like structures and localized bleeding

The evidence presented thus far in support of the thesis that *Wnt7a*, *Wnt7b*, *Gpr124* and *Reck* influence a common signaling pathway in limb development is based on skeletal phenotypes. If this thesis is correct, one might predict that non-skeletal phenotypes would also be shared across different combinations of mutant alleles, subject to the constraint that some phenotypes might only manifest when the strength of the β-catenin signal is reduced below a criterion threshold. We have explored this line of thinking with two macroscopically observable non-skeletal phenotypes: the growth of ectopic nail-like structures, observable in postnatal mice, and the presence of localized bleeding in a digit, observable in embryos.

**Fig. 7.**
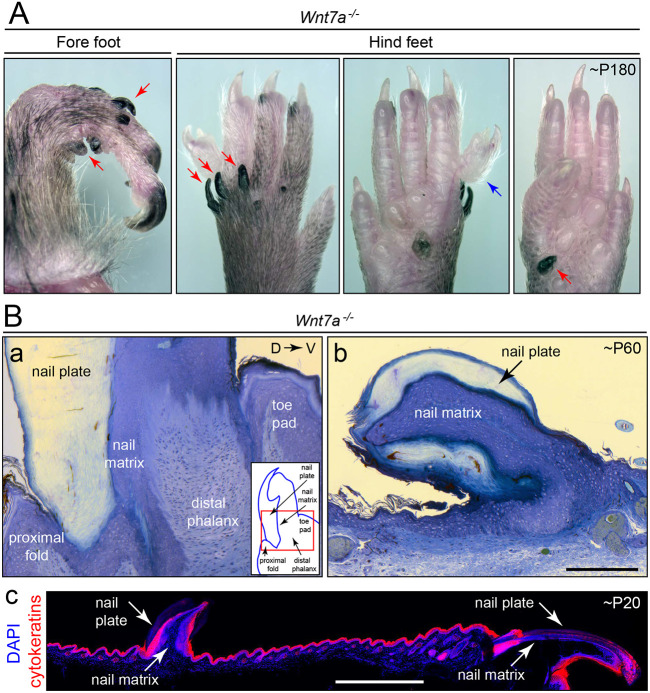
***Wnt7a* loss causes the growth of ectopic nail-like structures.** (A) Ectopic nail-like structures growing on the dorsal (and, rarely, on the ventral) surfaces of fore and hind feet of *Wnt7a^−/−^* mice at ∼P180. Dorsal (left) and ventral (right) views of one hind foot are shown in the central two panels. (B) Panels Ba and Bb show Methylene Blue-stained sections of epon-embedded feet at ∼P60 showing a normal nail bed (Ba) and an ectopic nail-like structure on the dorsal surface of the foot (Bb). Inset in Ba shows nail bed anatomy, with the region shown in the image in Ba enclosed in the red rectangle. Panel Bc shows a frozen section through the dorsal surface of a digit at ∼P20, immunostained with mAb AE13, which recognizes hair and nail cytokeratins. The true nail/claw is at the right side of the image and an ectopic nail-like structure is on the left side. Scale bars: 200 μm (Ba,Bb); 1 mm (Bc).

Ectodermal protrusions, mostly located on the dorsal surface of the feet, have been previously noted in *Wnt7a* and *Reck* mutant mice and have been variously identified as ectopic footpads (i.e. the pads that normally develop on the ventral surface of the foot; Parr and McMahon, 1995; Parr et al., 1998), pigmented nail-like structures (Kimura et al., 2010) or cutaneous horns (Yamamoto et al., 2012). Fig. 7A shows the gross appearance of these structures and the various locations at which they arise in *Wnt7a^−/−^* mice. In cross-section, these nail-like structures contain a central nail matrix flanked by nail plates, and they secrete one or more cytokeratins found in hair and other ectodermal structures (recognized by the pan-cytokeratin mAb AE13; Fig. 7B). Importantly, ectopic nail-like structures are also present on the hind feet of *Cdx2-Cre;Gpr124^CKO/−^;Reck^PW/CKO^* mice (Fig. 5A), with the same general appearance and range of locations as seen on both fore and hind feet in *Wnt7a^−/−^* mice.

Bleeding into a digit was observed in a subset of hind feet in affected embryos and was correlated with phenotypic severity (red arrows in Figs 3A, 4A, and Figs S1 and S3). Bleeding was present in 0% (0/67) of *Wnt7a^−/−^* embryos, 5% (1/19) of *Cdx2-Cre;Wnt7a^−/−^;Wnt7b^CKO/−^* embryos, 6% (1/17) of *Msx2-Cre;Wnt7a^−/−^;Wnt7b^CKO/−^* embryos and 36% (4/11) of *Cdx2-Cre;Wnt7a^−/−^;Tbx3^CKO/−^* embryos. The frequency of digit bleeding was increased in the allelic combinations of *Wnt7a* plus *Reck* and *Wnt7a* plus *Gpr124* that showed greater severities of skeletal defects. Specifically, digit bleeding was present in 100% (12/12) of *Wnt7a^−/−^;Reck^Δex2/Δex2^* embryos, compared with 7% (1/14) of *Reck^Δex2/Δex2^* embryos. Similarly, digit bleeding was present in 86% (12/14) of *Wnt7a^−/−^;Gpr124^−/−^* embryos, compared with 0% (0/35) of *Gpr124^−/−^* embryos. Finally, in the *Reck* allelic combination with the most severe skeletal defects (*Reck^Δex2/PW^*), digit bleeding was present in 52% (13/25) of embryos. By contrast, it was absent in *Reck^Δex2/Δex2^* embryos (0/19 embryos), which have a milder phenotype. Bleeding in the brain and spinal cord is seen in *Gpr124^−/−^* and *Reck^Δex2/Δex2^* embryos (Zhou and Nathans, 2014; Cho et al., 2017), but its mechanism and relationship to defective angiogenesis and barrier formation is currently unknown. Bleeding into a digit may represent an analogous process in the peripheral vasculature.

These data indicate that repressing a nail-like developmental program in the ectoderm and maintaining the integrity of digit blood vessels are dependent on the actions of *Wnt7a*, *Wnt7b*, *Gpr124* and *Reck*, further supporting the thesis that these four genes act in concert in the developing limb. Whether the growth of nail-like structures and the bleeding phenotype reflect a direct role for β-catenin signaling in the relevant cell types or are secondary to signaling events in adjacent tissues – for example, limb mesoderm derivatives – is, at present, unknown.

### LMX1B localization in WT versus mutant limbs

In previous work, the transcription factor LMX1B has been shown to function as one of the master-regulators of dorsal-ventral patterning in the limb (Riddle et al., 1995; Vogel et al., 1995; Cygan et al., 1997; Chen et al., 1998; Li et al., 2010). During limb development, dorsal ectoderm-derived WNT7A induces and maintains *Lmx1b* expression in the dorsal mesenchyme. Loss of *Lmx1b* leads to ventralization of the dorsal limb and ectopic expression of *Lmx1b* in the ventral mesenchyme leads to dorsalization of the ventral limb. Here, we extend these earlier observations by immunostaining for LMX1B in E13 limbs from WT, *Wnt7a^−/−^*, *Wnt7a^−/−^;Wnt7b^+/−^* and *Reck^Δex2/PW^* embryos.

**Fig. 8.**
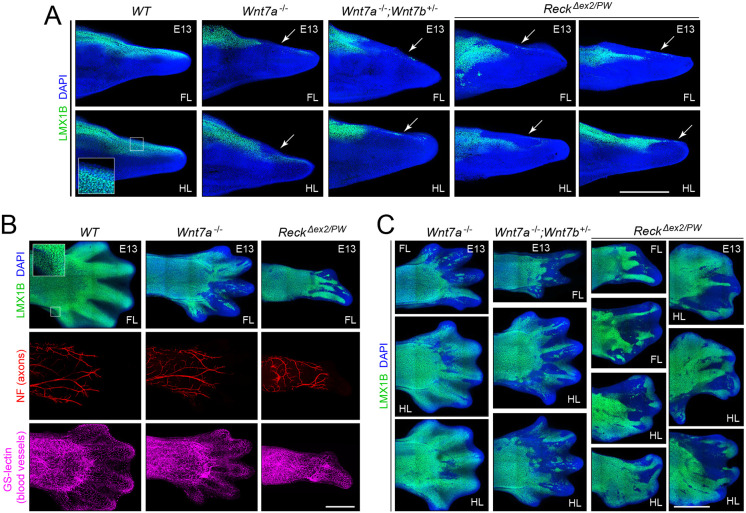
**Spatial distribution of LMX1B in WT and mutant limbs at E13.** (A) Immunostaining of vibratome sections showing the distal region of E13 limbs from embryos of the indicated genotypes. Dorsal is up. The inset in the leftmost panel is an enlargement of the boxed area and shows the nuclear localization of LMX1B. White arrows point to the distal-dorsal region of the mutant limbs with reduced or absent LMX1B expression. (B) Whole E13 limbs stained with anti-LMX1B, anti-neurofilament (NF; nerve fibers) and GS-lectin (blood vessels). The immunostained nerve fibers, which are present in the limb interior, demonstrate the accessibility of the entire limb to the antibody probes. Blood vessels, visualized with GS-lectin, are present throughout the limb. The inset in the leftmost panel is an enlargement of the boxed area and shows the nuclear localization of LMX1B. (C) Additional whole limb immunostaining, as in B, showing the large-scale spatial arrangement of LMX1B-positive and LMX1B-negative cells in the indicated mutant limbs. All limbs in C were co-stained with anti-NF and GS-lectin to demonstrate probe access to the limb interior. DAPI staining (blue) shows the locations of nuclei. FL, fore limb; HL, hind limb. Scale bars: 0.5 mm.

Sections of WT limbs in the plane defined by the proximal-distal and dorsal-ventral axes showed LMX1B within dorsal nuclei, a pattern that extended to within tens of microns of the limb tip (Fig. 8A). In *Wnt7a^−/−^* limbs, LMX1B was markedly reduced in the distal ∼500 μm of the limb. Similar reductions were observed in *Wnt7a^−/−^;Wnt7b^+/−^* and *Reck^Δex2/PW^* limbs, but in all three mutant genotypes the reduced expression was spatially heterogeneous, with variable loss of LMX1B at different depths within the limb and small clusters of LMX1B-positive nuclei often observed within distal limb regions (Fig. 1A). To obtain a more holistic view of LMX1B localization, intact E13 limbs were immunostained and imaged from the dorsal side (Fig. 8B,C). Simultaneous immunostaining for neurofilament (NF) revealed the growing nerve plexus in the limb interior and served as a technical control for antibody penetration throughout the limb (Fig. 8B). Loss of LMX1B was observed in a spatially patchy distribution, with *Wnt7a^−/−^* limbs showing a more limited loss of LMX1B compared with *Wnt7a^−/−^;Wnt7b^+/−^* and *Reck^Δex2/PW^* limbs. *Reck^Δex2/PW^* limbs also showed the greatest degree of disorganization within the digit region. This pattern of spatial heterogeneity of LMX1B in the distal region of *Wnt7a^−/−^;Wnt7b^+/−^* and *Reck^Δex2/PW^* limbs, but not in WT limbs, was also observed at E11 (white arrowheads in Fig. S4). At E11, co-staining for LEF1, a Wnt co-activator and the product of a Wnt-activated gene, reveals similar truncations of the limb bud on the side away from the thumb in both *Wnt7a^−/−^;Wnt7b^+/−^* and *Reck^Δex2/PW^* embryos, with fore limbs more affected than hind limbs (white arrowheads in Fig. S4). These observations add further support to a model in which RECK acts within the same pathway as WNT7A and WNT7B during limb development. They also suggest that under conditions of reduced β-catenin signaling, dorsal mesoderm cells respond in a heterogeneous manner, with clusters of neighboring cells differing markedly in the level of *Lmx1b* expression.

## DISCUSSION

The genetic analyses presented here show that a distinctive set of skeletal, ectodermal and vascular phenotypes in the mouse limb are made progressively more severe with the step-wise inactivation of various combinations of *Wnt7a*, *Wnt7b*, *Reck*, *Gpr124* and/or *Tbx3*. Taken together, these data support a model in which the WNT7A/WNT7B-FRIZZLED-LRP5/LRP6-GPR124-RECK signaling system, first defined in the context of CNS angiogenesis and barrier development, also functions as an integral unit in the developing limb, with the resulting β-catenin signal converging on a TBX3-containing transcription complex and regulating LMX1B. This work adds to a broad and recurring theme in the field of cell-cell signaling: that nature repurposes a relatively small number of signaling systems – including ligands, receptors, and regulators – to control a wide diversity of developmental processes. The diversity of transcriptional outputs associated with β-catenin signaling in different developmental contexts presumably reflects different chromatin and transcription factor landscapes within the target cells, as shown by ChIP-seq and ATAC-seq (e.g. Bottomly et al., 2010; Estarás et al., 2015; Sabbagh et al., 2018; Eray et al., 2020).

The present work reveals an intriguing difference between the effects of *Gpr124* and *Reck* loss-of-function in CNS vascular versus limb development. In the context of CNS angiogenesis, *Gpr124*, *Reck* or *Wnt7a* plus *Wnt7b* loss-of-function leads to severe and roughly similar phenotypic effects (Stenman et al., 2008; Daneman et al., 2009; Chandana et al., 2010; Kuhnert et al., 2010; Anderson et al., 2011; Cullen et al., 2011; Zhou and Nathans, 2014; Posokhova et al., 2015; Vanhollebeke et al., 2015; Cho et al., 2017), whereas, in the developing limb, *Gpr124* loss-of-function leads to a substantially milder phenotype than either *Reck* or *Wnt7a* plus *Wnt7b* loss-of-function. However, in contrast to this *Gpr124* versus *Reck* difference in phenotypic severity, when *Gpr124* or *Reck* loss-of-function mutations are combined with heterozygous or homozygous *Wnt7a* loss-of-function – i.e. when the WNT7A/WNT7B signal is reduced and is made more dependent on WNT7B – the additional loss of *Gpr124* or *Reck* produces similarly strong enhancements of the limb phenotype (Figs 2 and 3). A general explanation for non-linearities in WNT7A/WNT7B signaling phenotypes and the different degree of dependence on GPR124 versus RECK function could be that, under WT conditions, WNT7A/WNT7B signaling is substantially above the threshold for normal limb development and that loss of GPR124 causes a more modest decrement in signal strength compared with loss of RECK. With reduced WNT7A/WNT7B signaling, the system may be sensitized to further loss in signal strength resulting from loss of either GPR124 or RECK. Analogous non-linear effects on CNS barrier integrity have been observed with different combinations of loss-of-function mutations in WNT7A/WNT7B and NORRIN signaling components (Wang et al., 2018). One mechanistic explanation for the apparently greater reliance of WNT7A/WNT7B signaling on RECK than on GPR124 could relate to the additional role that RECK plays in WNT7A/WNT7B trafficking, with RECK apparently acting as a chaperone to increase the transport of bioactive WNT7A and WNT7B (Li et al., 2019; Martin et al., 2022).

The spatial heterogeneity of LMX1B expression among cells in the distal limb in *Wnt7a^−/−^*, *Wnt7a^−/−^;Wnt7b^+/−^*, and *Reck^Δex2/PW^* embryos is reminiscent of the heterogeneity among CNS ECs in their response to reduced β-catenin signaling. With step-wise reductions in β-catenin signaling, greater numbers of CNS ECs convert from a BBB competent state to a non-barrier state. At the level of individual ECs, the patterns of gene expression appear to be largely quantized, with cells adopting either a barrier-competent state or a non-barrier state (Zhou et al., 2014; Wang et al., 2018). The recent discovery of positive feedback in *Lmx1b* transcription via auto-regulatory enhancers may explain, at least in part, the quantization of LMX1B levels in cells that receive a reduced β-catenin signal (Haro et al., 2021). To account for the spatial clustering of *Lmx1b*-expressing versus non-expressing cells, one would additionally need to postulate a feedback process at the level of cell-cell communication.

A still unresolved question is how WNT signaling is orchestrated in space and time in structures, such as the limb and brain, that express multiple WNTs and FRIZZLEDs in substantially overlapping patterns (Shimogori et al., 2004; Fischer et al., 2007; Summerhurst et al., 2008; Witte et al., 2009). Cell culture experiments that measured signaling by each of the 190 pairwise combinations of 10 FRIZZLEDs and 19 WNTs showed substantial ligand-receptor promiscuity (Yu et al., 2012), consistent with crystal structures of WNT bound to the FRIZZLED cysteine-rich-domain that show protein-protein contacts confined to two relatively small surfaces that are composed of mostly conserved amino acids (Janda et al., 2012; Hirai et al., 2019). *In vivo*, partial redundancy is observed for subsets of Frizzled genes (e.g. *Fzd1*/*Fzd2*/*Fzd7*, *Fzd3*/*Fzd6* and *Fzd4*/*Fzd8*) (Wang et al., 2016), although it is unclear whether this functional redundancy reflects promiscuity between WNTs and FRIZZLEDs, additivity of β-catenin signal strength mediated by distinct WNT-FRIZZLED pairs or a combination of both effects.

In contrast with the partial redundancy described in the preceding paragraph, there are numerous examples of single Frizzled KO and single Wnt KO phenotypes in tissues where multiple Frizzled or Wnt family members are expressed in partially or largely overlapping patterns (van Amerongen and Berns, 2006; Wang et al., 2016; http://web.stanford.edu/group/nusselab/cgi-bin/wnt/). In the present example, loss of *Wnt7a* and *Wnt7b* in the limb leads to severe skeletal phenotypes despite the expression of other Wnt genes – including *Wnt3*, *Wnt4* and *Wnt6* – in essentially the same ectodermal pattern (Summerhurst et al., 2008; Witte et al., 2009). These observations imply a substantial degree of WNT-FRIZZLED specificity *in vivo*.

For WNT7A and WNT7B, one source of this specificity has emerged with the identification of GPR124 and RECK as ligand-specific co-activators, initially in the context of CNS vascular development and, with the present work, in the context of limb development. For the non-WNT ligand NORRIN, the tetraspanin TSPAN12 plays an analogous role (Junge et al., 2009; Lai et al., 2017). Auxiliary proteins that modulate ligand-receptor specificity have also been identified for a variety of G-protein coupled receptors and ligand-gated ion channels (Kato et al., 2010; Pinard et al., 2010; Galaz et al., 2015; Serafin et al., 2020). It will be interesting to determine whether additional specificity factors exist in the WNT-FRIZZLED system.

## MATERIALS AND METHODS

### Mouse lines and husbandry

The following mouse lines were used: *Msx2-Cre* (Sun et al., 2000; JAX 027892, the Jackson Laboratory); *Prx1-Cre* (Logan et al., 2002; also called *Prrx1-Cre*; JAX 005584, the Jackson Laboratory); Cdx2-Cre (Hinoi et al., 2007; JAX 009350, the Jackson Laboratory); *Gpr124^CKO^* and *Gpr124^KO^* (Posokhova et al., 2015; JAX 016881, the Jackson Laboratory); *Wnt7a^CKO^* (a gift from Thomas Spencer, University of Missouri, MO, USA); *Wnt7a^KO^* (Parr and McMahon, 1995; JAX 004715, the Jackson Laboratory); *Wnt7b^CKO^* (Rajagopal et al., 2008; JAX 008467, the Jackson Laboratory); *Wnt7b^KO^* (Parr et al., 2001; JAX 004693, the Jackson Laboratory); *Tbx3^CKO^* and *Tbx3^KO^* (Frank et al., 2012; a gift from Dr Anne Moon, Geisinger Commonwealth School of Medicine, PA, USA); *Reck^Δex2^* (Chandana et al., 2010; a gift from Dr Makoto Noda, Kyoto University, Japan); *Reck^PW^* (Cho et al., 2019; JAX 033395, the Jackson Laboratory). All mice were housed and handled according to the approved Institutional Animal Care and Use Committee protocol of the Johns Hopkins Medical Institutions.

### Scoring limb defects

For embryo skeletons, limb phenotypes were assigned a score as follows: one point for each missing digit and two points for each missing distal limb bone (fibula or ulna). For postnatal mice, limb phenotypes, which were relatively mild, were assigned one point for each missing digit. *P*-values were calculated using the non-parametric Mann–Whitney–Wilcoxon.

### μCT

Fore limbs and hind limbs were dissected and evaluated using a SkyScan 1275 high-resolution μCT imaging system (Bruker). Each fore limb or hind limb was scanned separately at 65 kV and 153 μA with a 1.0-mm aluminum filter to obtain a 15-μm voxel size, exposure time of 160-218 ms, frame averaging of 4 and rotation step of 0.3 degrees. NRecon (Bruker) was used to reconstruct images with the following settings: ring artifact reduction of 5% and beam-hardening correction of 20%. CTVox (v3.2, Bruker) was used to generate the 3D morphometric analyses of images.

### Skeleton preparations

E14.5-E16.5 embryos were fixed in Bouin's fixative (Sigma-Aldrich, HT10132-1L) at room temperature overnight on a horizontal shaker. The fixed embryos were washed >10 times in 70% ethanol/1% NH_4_OH over the course of 24 h, until they appeared white. The embryos were washed twice in 5% acetic acid, 1 h each, followed by overnight incubation at room temperature in Alcian Blue solution [80% ethanol, 20% acetic acid, 150 mg/l Alcian Blue 8GX (Sigma-Aldrich, 5268-25G)]. Stained embryos were washed twice in 5% acetic acid, 1 h each, followed by two washes in 95% ethanol, 1 h each. Dehydrated embryos were cleared and stored in BBBA solution [2:1 benzyl benzoate (Sigma-Aldrich, B6630-1L): benzyl alcohol (Sigma-Aldrich, 402834-1L)] in 20 ml glass scintillation vials.

### Epon sections of adult feet

Starting with feet at around postnatal day (P)60, incisions were made in the ventral skin and the bones were removed. The bone-free tissues were fixed in 2% paraformaldehyde (PFA) +2% glutaraldehyde in PBS overnight at 4°C, and then treated for 90 min with 1% osmium tetroxide on ice, dehydrated in an ethanol series, embedded in Epon, sectioned at 0.5 μm thickness and stained with Methylene Blue.

### Antibodies

The following antibodies were used at the indicated dilutions: AF594-conjugated pan-cytokeratin mouse mAb AE13; (1:400; sc-57012, Santa Cruz Biotechnology); guinea pig anti-LMX1B (1:20,000; a gift from Dr Thomas Mueller; Müller et al., 2002); chicken anti-neurofilament heavy chain (1:400; NFH, Aves Labs); rabbit anti-LEF1 (1:400; C12A5, Cell Signaling Technology). Alexa Fluor-conjugated secondary antibodies were from Thermo Fisher Scientific (anti-chicken Alexa 594, A11042; anti-guinea pig Alexa 543, A11076; anti-mouse Alexa 594, A11005; and anti-rabbit Alexa 594, A11012) and used at 1:400. Alexa647-conjugated Isolectin GS-IB4 was from Thermo Fisher Scientific (I32450) and used at 1:400.

### Immunostaining

Eighteen-micron-thick sections of fresh frozen limbs were cut on a cryostat (Zeiss Microm HM500M) from OCT blocks and stained with primary antibodies diluted in 7% normal goat serum (NGS) in PBSTC (PBS+0.1 mM CaCl_2_+1% TritonX-100) overnight at 4°C. The sections were washed in PBSTC three times at room temperature, each for 10 min. The sections were subsequently incubated in fluorescent secondary antibodies (1:400) in 7% NGS in PBSTC at room temperature for 2 h, washed in PBSTC three times at room temperature, each for 10 min, and mounted in Fluoromount-G (SouthernBiotech, 0100-01).

For immunostaining of intact E13 limbs, decapitated E13 embryos were fixed in 1% PFA overnight at 4°C. Limbs with attached flanks (to assist in the identification of each limb) were dissected from the embryos, and incubated in PBSTC overnight at 4°C. The limbs were then incubated in primary antibodies diluted in 7% NGS in PBSTC, at 4°C for 2 days. The limbs were washed in PBSTC for 8 h with hourly change of solution, followed by 2 days of incubation in secondary antibodies diluted in 7% NGS in PBSTC. Finally, the limbs were washed extensively in PBSTC before being flat-mounted in Flouromount-G.

For vibratome section immunostaining, decapitated E13 embryos fixed in 1% PFA overnight at 4°C were further incubated in methanol overnight at 4°C to increase tissue firmness for easier sectioning. The embryos were then rehydrated in PBS for 3 h at room temperature. Limbs were dissected and embedded in 3% low-melting-point agarose and sectioned longitudinally with a Leica vibratome (Leica VT1200) at 100 μm thickness. The immunostaining protocol for vibratome sections was identical to that for the intact limbs, except that the incubation times for the primary and secondary antibodies was 1 day instead of 2 days.

### Confocal microscopy

Confocal images were captured using a Zeiss LSM700 confocal microscope using Zen software, and processed with Adobe Photoshop and Adobe Illustrator software.

## Supplementary Material

Supplementary information

Reviewer comments
