# Supplementary material for: The WNT7A/WNT7B/GPR124/RECK signaling module plays an essential role in mammalian limb development
Source: Development. 2022 May 12;149(9):dev200340. doi: 10.1242/dev.200340 (PMC9148564; doi:10.1242/dev.200340)
Supplement: Supplementary information [file develop-149-200340-s1.pdf]

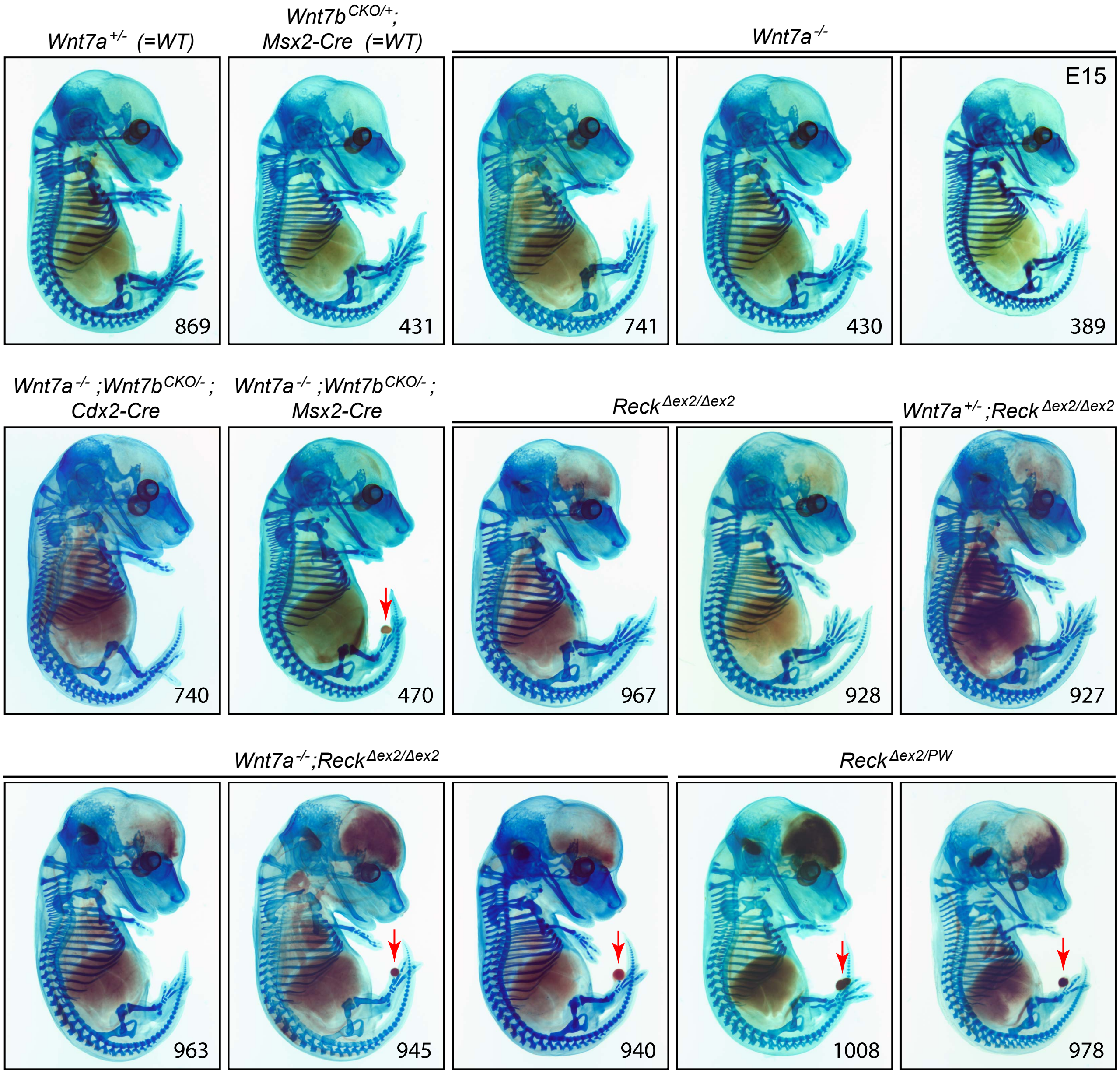

Fig. S1. E15 whole skeleton analysis for select mutant combinations of *Wnt7a*, *Wnt7b*, and *Reck* genes. Embryos were stained with Alcian Blue. Red arrows indicate regions with local bleeding, a characteristic of some allelic combinations with the most severe morphological defects. One upper and one lower limb have been removed from each embryo to assist in visualizing the remaining limb.

Development • Supplementary information

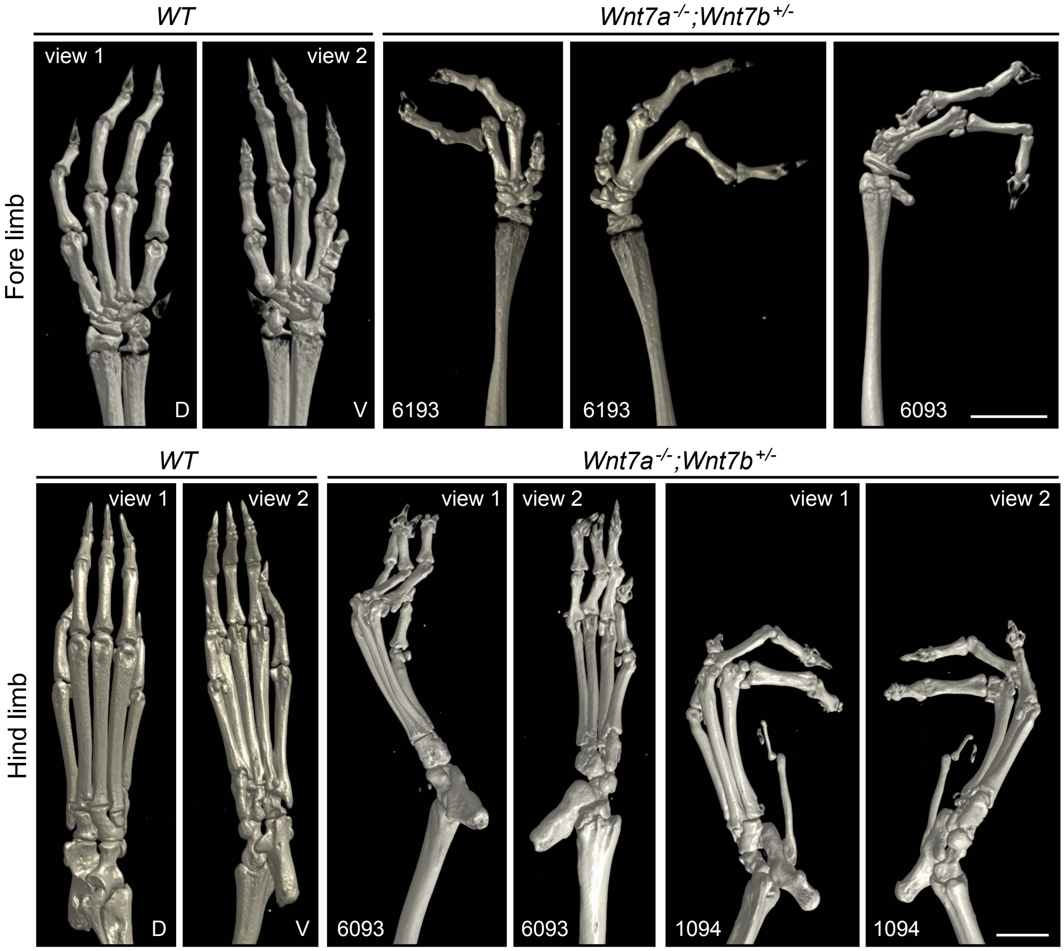

Fig. S2. Skeletal defects in the feet of *Wnt7a*$^{-/-}$;*Wnt7b*$^{+/-}$ mice. uCT of the distal regions of the fore and hind limbs from ~6 month old WT mice (left two panels) and *Wnt7a*$^{-/-}$;*Wnt7b*$^{+/-}$ mice (right panels). *Wnt7a*$^{-/-}$;*Wnt7b*$^{+/-}$ fore limbs show more severe defects than hind limbs, including fusion of the radius and ulna, and loss and/or fusion of carpal and metacarpal bones. Two views are shown for each WT limb, for the leftmost *Wnt7a*$^{-/-}$;*Wnt7b*$^{+/-}$ fore limb, and for both *Wnt7a*$^{-/-}$;*Wnt7b*$^{+/-}$ hind limbs. D, dorsal view. V, ventral view.

Development • Supplementary information

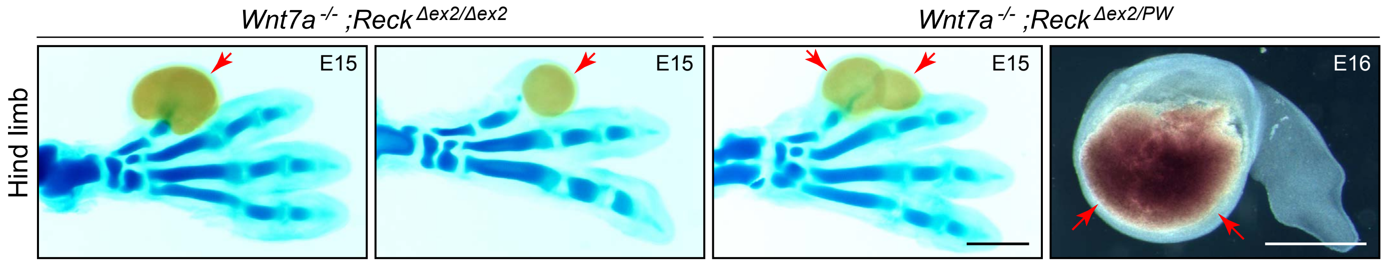

Fig. S3. Enlarged view of digit bleeding (red arrows) in Alcian Blue and BBBA-cleared E15 hind limbs (left three panels) and in a vibratome section of the digit region of an E16 hind limb (right). Note that BBBA leads to tissue shrinkage to ~60-70% of the original linear dimensions. Scale bars, 0.5 mm.

Development • Supplementary information

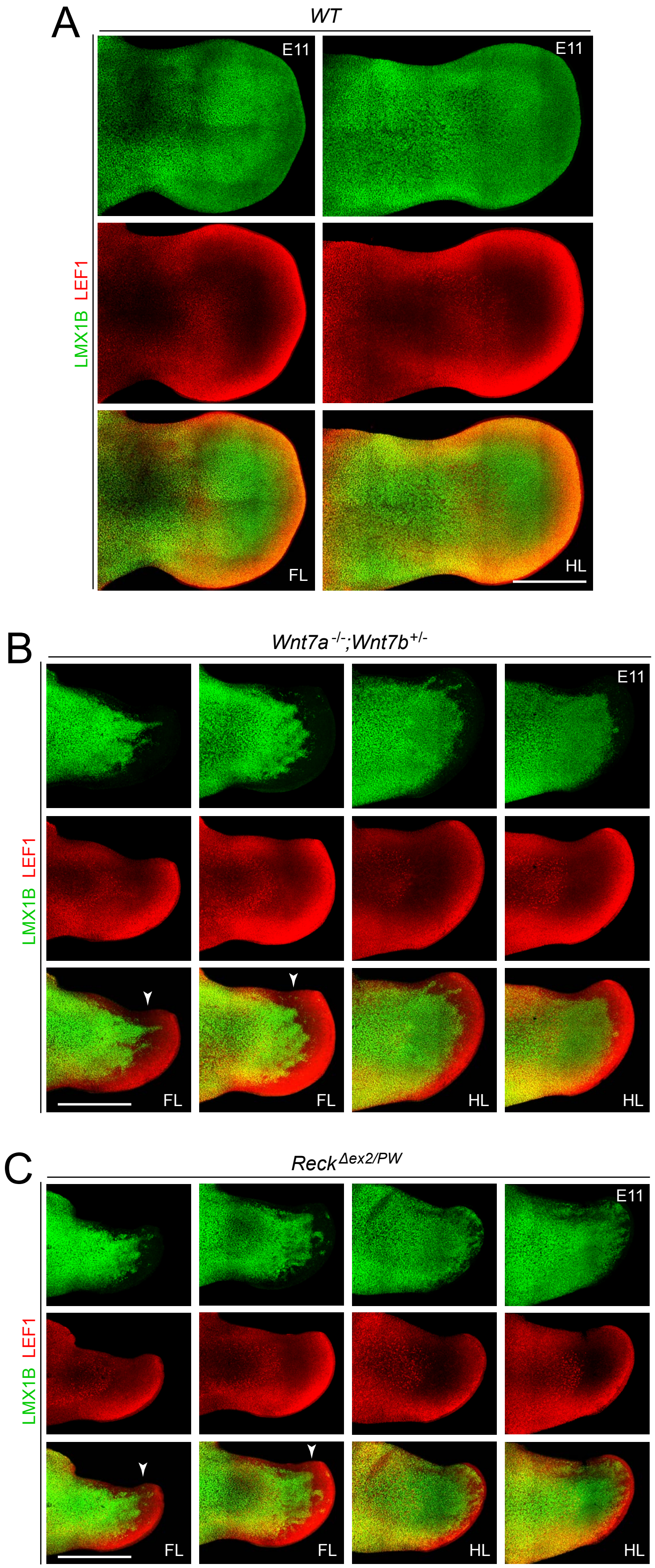

Development • Supplementary information

Fig. S4. Spatial distribution of LMX1B in WT and mutant limbs at E11. Whole E11 limbs were stained with anti-LMX1B and anti-LEF1. (A) Wild type; (B) *Wnt7a*$^{-/-}$;*Wnt7b*$^{+/-}$; and (C) *Reck* $^{\Delta ex2/PW}$. In (B) and (C), white arrowheads point to the distal region of the mutant limbs with spatially heterogeneous LMX1B accumulation and truncation of the limb bud on the side opposite the thumb. Images of right limbs have been inverted about a vertical axis for ease of comparison with images of left limbs. FL, forelimb. HL, hindlimb. Scale bars, 0.5 mm.

Table S1. The number of embryos stained with Alcian Blue and analyzed for skeletal defects, as presented in Figure 1-4 and 6, organized by figure and genotype.

Genotype	Number of embryos
Figure 1	
$Wnt7a^{+/-};Wnt7b^{+/-}$	27
$Cdx2\text{-}Cre;Wnt7a^{+/-};Wnt7b^{CKO/-}$	3
$Msx2\text{-}Cre;Wnt7a^{+/-};Wnt7b^{CKO/-}$	7
$Wnt7a^{-/-};Wnt7b^{+/+}$	26
$Wnt7a^{-/-};Wnt7b^{CKO/+}$	12
$Cdx2\text{-}Cre;Wnt7a^{-/-};Wnt7b^{CKO/+}$	24
$Msx2\text{-}Cre;Wnt7a^{-/-};Wnt7b^{CKO/+}$	12
$Wnt7a^{-/-};Wnt7b^{+/-}$	35
$Wnt7a^{-/-};Wnt7b^{CKO/-}$	40
$Cdx2\text{-}Cre;Wnt7a^{-/-};Wnt7b^{CKO/-}$	19
$Msx2\text{-}Cre;Wnt7a^{-/-};Wnt7b^{CKO/-}$	17
Figure 2	
$Wnt7a^{+/+};Gpr124^{+/+}$	5
$Wnt7a^{+/+};Gpr124^{+/-}$	7
$Wnt7a^{+/+};Gpr124^{-/-}$	36
$Wnt7a^{+/-};Gpr124^{+/-}$	16
$Wnt7a^{+/-};Gpr124^{-/-}$	14
$Wnt7a^{-/-};Gpr124^{+/+}$	11
$Wnt7a^{-/-};Gpr124^{+/-}$	23
$Wnt7a^{-/-};Gpr124^{-/-}$	14
Figure 3	
$Wnt7a^{+/+};Reck^{\Delta ex2/+}$	7
$Wnt7a^{+/+};Reck^{\Delta ex2/\Delta ex2}$	14
$Wnt7a^{+/-};Reck^{+/+}$	10
$Wnt7a^{+/-};Reck^{PW/+}$	13
$Wnt7a^{+/-};Reck^{\Delta ex2/\Delta ex2}$	17
$Wnt7a^{-/-};Reck^{+/+}$	16
$Wnt7a^{-/-};Reck^{\Delta ex2/+}$	31
$Wnt7a^{-/-};Reck^{PW/+}$	5
$Wnt7a^{-/-};Reck^{\Delta ex2/\Delta ex2}$	12
Figure 4	
$Gpr124^{+/+};Reck^{+/+}$	20
$Gpr124^{+/+};Reck^{\Delta ex2/+}$	17
$Gpr124^{+/+}; Reck^{PW/+}$	12
$Gpr124^{+/+};Reck^{\Delta ex2/\Delta ex2}$	25
$Gpr124^{+/+};Reck^{\Delta ex2/PW}$	25
$Gpr124^{+/-};Reck^{+/+}$	16
$Gpr124^{+/-};Reck^{\Delta ex2/\Delta ex2}$	7
$Gpr124^{-/-};Reck^{+/+}$	36
$Gpr124^{-/-};Reck^{\Delta ex2/+}$	9
$Gpr124^{-/-}; Reck^{PW/+}$	17

Development • Supplementary information

Genotype	Number of embryos
Figure 6	
Cdx2-Cre;Tbx3$^{CKO/-}$*;Wnt7a*$^{+/+}$	27
Cdx2-Cre;Tbx3$^{CKO/+}$*;Wnt7a*$^{+/-}$	8
Tbx3$^{+/-}$*;Wnt7a*$^{+/-}$	13
Cdx2-Cre;Tbx3$^{CKO/-}$*;Wnt7a*$^{+/-}$	28
Wnt7a$^{-/-}$	31
Cdx2-Cre;Tbx3$^{CKO/+}$*;Wnt7a*$^{-/-}$	13
Tbx3$^{+/-}$*;Wnt7a*$^{-/-}$	30
Cdx2-Cre;Tbx3$^{CKO/-}$*;Wnt7a*$^{-/-}$	11
