# Supplementary material for: The WNT7A/WNT7B/GPR124/RECK signaling module plays an essential role in mammalian limb development
Source: Development. 2022 May 12;149(9):dev200340. doi: 10.1242/dev.200340 (PMC9148564; doi:10.1242/dev.200340)
Supplement: Reviewer comments [file develop-149-200340_review_history.pdf]

The WNT7A/WNT7B/GPR124/RECK signaling module plays an essential role in mammalian limb development

Yanshu Wang, Arjun Venkatesh, Jiajia Xu, Mingxin Xu, John Williams, Philip M. Smallwood, Aaron J. James and Jeremy Nathans
DOI: 10.1242/dev.200340

Editor: Liz Robertson

Review timeline

Original submission:	8 November 2021
Editorial decision:	22 December 2021
First revision received:	10 March 2022
Editorial decision:	14 April 2022
Second revision received:	15 April 2022
Accepted:	20 April 2022

Original submission

First decision letter

MS ID#: DEVELOP/2021/200340

MS TITLE: The WNT7A/WNT7B/GPR124/RECK Signaling Module Plays an Essential Role in Mammalian Limb Development

AUTHORS: Jeremy Nathans, Yanshu Wang, Arjun Venkatesh, Jiajia Xu, Mingxin Xu, John Williams, Philip M. Smallwood, and Aaron James

I have now received all the referees' reports on the above manuscript, and have reached a decision. The referees' comments are appended below, or you can access them online: please go to BenchPress and click on the 'Manuscripts with Decisions' queue in the Author Area.

As you will see, the referees express considerable interest in your work, but have some significant criticisms and recommend a substantial revision of your manuscript before we can consider publication. If you are able to revise the manuscript along the lines suggested, which may involve further experiments, I will be happy receive a revised version of the manuscript. Your revised paper will be re-reviewed by one or more of the original referees, and acceptance of your manuscript will depend on your addressing satisfactorily the reviewers' major concerns. Please also note that Development will normally permit only one round of major revision.

We are aware that you may be experiencing disruption to the normal running of your lab that make experimental revisions challenging. If it would be helpful, we encourage you to contact us to discuss your revision in greater detail. Please send us a point-by-point response indicating where you are able to address concerns raised (either experimentally or by changes to the text) and where you will not be able to do so within the normal timeframe of a revision. We will then provide further guidance. Please also note that we are happy to extend revision timeframes as necessary.

Please attend to all of the reviewers' comments and ensure that you clearly highlight all changes made in the revised manuscript. Please avoid using 'Tracked changes' in Word files as these are lost in PDF conversion. I should be grateful if you would also provide a point-by-point response detailing

© 2022. Published by The Company of Biologists under the terms of the Creative Commons Attribution License (https://creativecommons.org/licenses/by/4.0/).

how you have dealt with the points raised by the reviewers in the 'Response to Reviewers' box. If you do not agree with any of their criticisms or suggestions please explain clearly why this is so.

Reviewer 1

Advance summary and potential significance to field

MS ID#: DEVELOP/2021/200340 MS title: The WNT7A/WNT7B/GPR124/RECK Signaling Module Plays an Essential Role in Mammalian Limb Development

This study uses several combinations of mutant alleles to study the effects of perturbation of Wnt ligands, Wnt7a and Wnt7b and components of the canonical Wnt transduction pathway, RECK and Gpr124, on limb development.

While a large number of mutant combinations have been generated and analysed, the overall conclusions drawn from the study do not represent a significant advance in understanding the role of the Wnt pathway in limb development.

Comments for the author

Several aspects of the presentation could be modified to improve clarity
The study uses a combination of different cre deleter lines to disrupt either the ectodermally-expressed Wnt ligands or the mesodermally-acting Wnt signal transduction components RECK/Gpr124. It is often not clearly described what is being tested with the various ectoderm-restricted (eg Msx2Cre) vs mesoderm-restricted (Prx1Cre) vs more ubiquitous (Cdx2Cre). Often the results of various combinations are gathered together in figures without the distinction of what has been tested being clearly expressed. In addition, the correct controls to compare with the mutants are not used throughout the study eg. Het conditional mutants over the cre delete allele. Much is left to the reader to decipher for themselves.
“bleeding into a digit” is not a sufficiently clear phenotype to score in this analysis. The observation is not elaborated on and therefore it is unclear what it may mean and if it is related to the other digit abnormalities described.
Many of the figures are extensive, containing many panels, but often with extraneous information. Eg. Figure 1 panel A does not provide clear information of the limb phenotypes-unprocessed whole embryos are shown, panel B-the number of the embryo shown is listed. This is not useful information to the reader and is likely to be confusing.
In Supplementary figure 1-The whole embryo shown. It would be better to show a magnified view of the isolated limb elements, as the limbs are not clearly visible.
Some of the mutant combinations give puzzling results In figure 1 it appears that presence of 1 copy of the Wnt7b CKO allele produced a higher phenotype score than +/+ even in the absence of any deleter transgenes. This is not discussed.
Of more concern, some aspects of the phenotypic presentation in the allelic series are hard to understand. One might expect if Gpr124 is required for Wnt7a/7b signaling then Gpr124 -/- should be as potent as a 7a/7b double KO, but this is apparently not the case from the results presented.
Some of the numbers of animals analysed are extremely high- > 1,000 Figure 5 is quoted (p3). It is hard to understand how this number is justifiable.
The connection between the analysis of the Wnt signalling components and Tbx3 is not clearly described. The ChIP seq data referenced does not prove this to be the case. The (common?) ulna phenotype might be of interest/relevance, but this is not discussed in detail.
A further interesting observation is the patches of clusters of cells with different Lmx1b expression, but this is not expanded upon.
The justification for scoring one point for a missing digit and 2 points for a missing fibula/ulna is unclear.

© 2022. Published by The Company of Biologists under the terms of the Creative Commons Attribution License (https://creativecommons.org/licenses/by/4.0/).

Reviewer 2

Advance summary and potential significance to field

Wang and colleagues dissect the contribution of WNT7A, WNT7B, RECK, and GPR124 in limb development through comprehensive gene dosage experiments. The authors thoroughly characterize combinations of hypomorphic, null, and conditional alleles of Wnt7a, Wnt7b, Reck, and Gpr124. Bone architecture, ectopic growth of nail-like structures, and marker gene expression are investigated in embryonic and adult mice. Overall, the data quality is very high (a strength of the study lies in its comprehensive nature). The paper is very well written, and the findings are interesting. The physiological role of this signaling axis has so far mainly been detailed in CNS vascular endothelial cells. Identifying a novel developmental setting controlled by RECK and GPR124 represents an important finding. We, therefore, recommend publication in Development, provided that the following comments are considered:

Comments for the author

Major comments:
1. In its present form, the paper does not provide direct evidence that RECK and GPR124 control limb development by activating beta-catenin signaling.
Conditional restoration of a constitutively-active beta-catenin allele and/or the analysis of Wnt activity reporter mice or staining for established Wnt target genes should be attempted (e.g. Axin2, Lef1). This is particularly important as GPR124 and RECK seem to have different impacts on limb development.
Direct analysis of Wnt activity might explain such differences.
2. Reduced Reck has been shown to downregulate Wnt7a expression in the forelimb at E11.5 (Yamamoto et al. 2012). Based on microarray evidence, ~35% reduction in Reck expression triggers a similar ~35% reduction in Wnt7a mRNA levels. This reduction seems even more drastic when assessed by ISH. Some of the Reck phenotypes reported in this paper could thus be a consequence of reduced expression of the ligand rather than the co-receptor itself. For example, the data presented in Fig. 3 are difficult to interpret without a direct measure of Wnt7a and Wnt7b expression level and distribution. This question should be addressed experimentally by probing the distribution of Wnt7a and Wnt7b mRNA in a selected set of genetic conditions (RT-PCR, ISH, RNAscope, immunostaining).
Data interpretation should be adjusted accordingly.
3. The data reveal that Wnt7a controls limb development at least partially independently of Gpr124, and maybe Reck. As a most striking illustration constitutive null Gpr124 mutants only exhibit mild phenotypes, that are exacerbated by the loss of Wnt7a (Fig. 2). The situation of Reck is less clear as conditional null alleles are not available in this study. However, a similar trend is noted in homozygous Reck hypomorphic mutants, which only exhibit modest developmental limb defects, that are exacerbated by Wnt7a loss of function (Fig. 3). Other Reck alleles combinations reveal stronger phenotypes (Fig. 4). The non-essential role of Gpr124 (and perhaps Reck) contrasts somewhat with the situation prevailing in the CNS, where the endothelial-specific loss of Gpr124 or Reck, more closely mirrors the consequences of Wnt7a/b combined deletion. We believe that the Gpr124 (and possibly Reck)-independent Wnt7a/b signaling occurring in the limb should be emphasized by the authors, as it might have important consequences on the interpretation of some key experiments (see the previous comment).
4. The parametric statistical test used throughout this study (Student's t-test)
is not suitable for analyzing the discrete (non-continuous) values of the embryonic limb phenotypic scoring system. Non-parametric analyses should be performed instead. This will likely affect some of the conclusions.

Minor comments:
1. Last paragraph of the introduction. We suggest detailing what the hypomorphic Reck mutants are (nature of the deletion, functional consequence).
2. Page 4. Please correct 'embryos have a skeletal defects'.

© 2022. Published by The Company of Biologists under the terms of the Creative Commons Attribution License (https://creativecommons.org/licenses/by/4.0/).

Reviewer 3

Advance summary and potential significance to field

In this manuscript titled "The WnT7A/WNT7B/GPR124/RECK signaling module plays an essential role in mammalian limb development", Wang and colleagues explored the genetic interactions between the canonical Wnt ligands WNT7A and WNT7B and their cell surface co-activators RECK and GPR124 in mouse limb development. This a tour de force study in which the authors used a large number of allelic combinations to demonstrate that WNT7A, WNT7B, RECK and GPR124 function in the same pathway to mediate canonical Wnt signaling and orchestrate proper limb development. This work complements previous studies showing that these molecules work in concert to media canonical Wnt signaling during angiogenesis, and further supports that the function of the WNT7/RECK/GPR124 signaling module is conserved across multiple tissue/organ systems. In addition, the study provides new insights on the broader questions of how signaling specificity is achieved within the Wnt signaling system, and how a relatively small number of developmental pathways can be reused and repurposed to drive diverse developmental processes. Overall, the quality of the study is excellent both in terms of its conceptual advancement and its experimental rigor.

Comments for the author

Addressing the comments below would improve the quality of the manuscript:
1. Can the authors mention how many animals were analyzed for the deformities as well as what the bars represent in the limb phenotype scoring analyses shown in Figs 1-4 (SE or SD)?
2. Some of the p values, particularly in Figure 4 seem unusually high. For example, the p value for Gpr124+/+; ReckΔ/Δ (blue) and Gpr124+/+; ReckΔ/PW (salmon) is mentioned as 2.7x10^-11, though the difference does not seem to approach that degree of significance. On the other hand, the comparison betweenGpr124+/-; Reck+/+ (light green) and Gpr124+/-; ReckΔ/Δ (dark green), which seems to be highly significant, has a p value of 6.4x10^-4. Can the authors provide clarifications on this?

First revision

Author response to reviewers' comments

We are grateful to you and the reviewers for your thoughtful and constructive suggestions to improve the manuscript. As a result, we have substantially expanded the text, added a new Supplemental Table 1, and added two new Supplementary Figures. In the paragraphs below, we reproduce the text of the reviewers' critiques and our point-by-point replies to those critiques. In the resubmitted article, text changes are in red. We have also included a figure showing snRNAseq UMAP plots that is not for publication.

Reviewer 1 Comments for the Author:
Several aspects of the presentation could be modified to improve clarity

#1 The study uses a combination of different cre deleter lines to disrupt either the ectodermally-expressed Wnt ligands or the mesodermally-acting Wnt signal transduction components RECK/Gpr124. It is often not clearly described what is being tested with the various ectoderm-restricted (eg Msx2Cre) vs mesoderm-restricted (Prx1Cre) vs more ubiquitous (Cdx2Cre). Often the results of various combinations are gathered together in figures without the distinction of what has been tested being clearly expressed. In addition, the correct controls to compare with the mutants are not used throughout the study eg. Het conditional mutants over the cre delete allele. Much is left to the reader to decipher for themselves.

REPLY. Thank you for that comment. We agree that the original text was relatively terse in its descriptions of the crosses and the controls. We have now expanded the Results section to make the descriptions more complete. Regarding controls for Cre lines, we have used Cre vs. no-Cre

© 2022. Published by The Company of Biologists under the terms of the Creative Commons Attribution License (https://creativecommons.org/licenses/by/4.0/).

comparisons (Figure 1), CKO/+ vs +/+ comparisons (Figures 5C), and CKO/+ vs CKO/- comparisons (Figure 6). Each of these three comparisons represent appropriate controls for the particular Cre crosses, and this is now more fully described in the Results section.

#2 “bleeding into a digit” is not a sufficiently clear phenotype to score in this analysis. The observation is not elaborated on and therefore it is unclear what it may mean and if it is related to the other digit abnormalities described.

REPLY. We have added a new Supplemental Figure 3 to show, at higher magnification, what the “bleeding into a digit” phenotype looks like in the Alcian Blue stained limbs and in a fixed and vibratome sectioned limb. The phenotype is striking and discrete – a limb either exhibits bleeding or it does not. That said, we do not have any mechanistic insight into the vascular defect(s) responsible for bleeding and we do not know how this phenotype relates mechanistically to the other limb phenotypes. It is intriguing that bleeding is part of the CNS vascular defect observed in Gpr124 KO and Reck KO embryos – this is now noted in the text. The important point in the context of the present work is that the frequency of the “bleeding into a digit” phenotype correlates closely with the severity of other limb phenotypes (e.g. bone defects) associated with genetic loss-of-function among all of the genes studied here and it is seen with multiple mutant combinations.

#3 Many of the figures are extensive, containing many panels, but often with extraneous information. Eg. Figure 1 panel A does not provide clear information of the limb phenotypes-unprocessed whole embryos are shown, panel B-the number of the embryo shown is listed. This is not useful information to the reader and is likely to be confusing.
In Supplementary figure 1-The whole embryo shown. It would be better to show a magnified view of the isolated limb elements, as the limbs are not clearly visible.

REPLY. We have removed the embryo numbers from each of the panels. We agree that this is extraneous information. Regarding Figure 1A, Figure 5B, and Supplemental Figure 1, we think that it is important to show the entire embryo and the entire skeleton in a few figures to show that the phenotypes (bone defects and localized bleeding) are confined to the limb, i.e. this is not a general skeletal development phenotype. Supplemental Figure 1 is a high resolution image, so that readers can enlarge the images and see the limbs at higher magnification. Figures 1-4 and 6 show magnified images of isolated limbs to visualize limb bone defects, and the new Supplemental Figure 3 shows magnified views of limbs with bleeding into a digit.

#4 Some of the mutant combinations give puzzling results. In figure 1 it appears that presence of 1 copy of the Wnt7b CKO allele produced a higher phenotype score than +/+ even in the absence of any deleter transgenes. This is not discussed.

REPLY. The reviewer is correct. In comparing the 4th and 5th genotypes plotted in Figure 1C, one would expect the Wnt7a-/-;Wnt7b+/+ and the Wnt7a-/-;Wnt7bCKO/+ phenotypes to be identical. Similarly, in comparing the 8th and 9th genotypes plotted in Figure 1C (Wnt7a-/-;Wnt7b+/- vs. Wnt7a-/-;Wnt7bCKO/-), on would expect identical phenotypes, but the latter group shows a slightly greater mean phenotype score. The simplest explanation for these discrepancies is that they arise from a modest reduction in Wnt7b expression from the CKO allele compared to the WT allele. We have added a sentence to the Figure1 legend describing this.

#5 Of more concern, some aspects of the phenotypic presentation in the allelic series are hard to understand. One might expect if Gpr124 is required for Wnt7a/7b signaling then Gpr124 -/- should be as potent as a 7a/7b double KO, but this is apparently not the case from the results presented.

REPLY. We thank the reviewer for this comment. It is clear that, in the limb, the Gpr124-/- phenotype is milder than either the Wnt7a-/-;Wnt7b-/- phenotype or the Reck mutant phenotype (compound heterozygote for Reck exon 2 deletion and the Reck PW point mutations). This observation is not a “concern” – it is real and it is interesting. We have now expanded the Discussion to comment on it. This question was also raised in comment #1 by reviewer #2.

A few additional comments on this topic:

© 2022. Published by The Company of Biologists under the terms of the Creative Commons Attribution License (https://creativecommons.org/licenses/by/4.0/).

1. The CNS vascular phenotype of Gpr124-/- embryos is quite severe, so clearly Gpr124 is important in this context. [An important detail: we (and others) do not have a side-by-side comparison between the Wnt7a and Wnt7b loss-of-function CNS phenotype vs. either the Reck or Gpr124 loss-of function phenotypes, because Wnt7a and Wnt7b loss-of-function phenotypes were analyzed by different research groups and because the Wnt7a and Wnt7b loss-of-function embryos die at ~E12.5, whereas Reck or Gpr124 mutant embryos survive until birth.]

2. Our genetic analyses in the CNS vasculature has provided evidence that there are dosage effects of Wnt/beta-catenin signaling components on the severity of the CNS vascular phenotype (Zhou, et al (2014) Canonical Wnt signaling components in vascular development and barrier formation. JCI 124: 3825-3846; Wang, et al (2018) Interplay of the Norrin and Wnt7a/Wnt7b signaling systems in blood-brain barrier and blood-retina barrier development and maintenance. PNAS 15: E11827-E11836). The present work demonstrates analogous dosage effects of Wnt/beta-catenin signal components on the severity of the limb phenotype.

3. In a luciferase reporter cell line, we have shown that expressing Reck and Gpr124 strongly potentiates Wnt7a/7b signaling, but there is still low level Wnt7a/7b signaling without Reck and Gpr124 over-expression – either because the Wnt-Frizzled-Lrp signaling complex can form at a reduced level independently of Reck and Gpr124 and/or because the reporter cells express low levels of Reck and Gpr124. So, it may be the case that, in vivo, Gpr124 and Reck potentiate Wnt7a/7b signaling but that there is still low level signaling in their absence.

In sum, the data imply that (1) Wnt7a/7b, Reck, and Gpr124 are playing major roles in beta-catenin signaling in CNS endothelial cells to control angiogenesis and barrier formation, (2) Wnt7a/7b and Reck are playing major roles in beta-catenin signaling in the limb, and (3) Gpr124 is playing a less important role in beta-catenin signaling in the limb. We have added a new paragraph to the Discussion to comment on this.

#6 Some of the numbers of animals analysed are extremely high- > 1,000 Figure 5 is quoted (p3). It is hard to understand how this number is justifiable.

REPLY. The >1,000 progeny mice described in Figure 5 derive from 8 different crosses, which are shown in Figure 5C and 5D. For the two crosses with the largest number of progeny (299 and 343 progeny), there were 8 possible genotypes among the progeny (the Cre transgene was heterozygous in the parent, as were Gpr124 and Reck), so we required many progeny to obtain a reasonable number of each genotypic class (see the histogram at the bottom of Figure 5D).

#7 The connection between the analysis of the Wnt signalling components and Tbx3 is not clearly described. The ChIP seq data referenced does not prove this to be the case. The (common?) ulna phenotype might be of interest/relevance, but this is not discussed in detail.

REPLY. We do not understand this comment. In the Results section, we introduce the Tbx3 experiment with a paragraph that summarizes the published evidence showing (1) that Tbx3 regulates limb development in mice and humans, and (2) that TBX3 is a component of the beta-catenin-LEF/TCF-BCL9 transcriptional regulatory complex. The experiments that we present in Figure 6 extend those observations by demonstrating a genetic interaction between Tbx3 and Wnt7a in mouse limb development. We have not discussed the ulna phenotype because we do not see that is being specific or distinctive – it merely represents the more extreme end of the phenotypic spectrum for reduced levels of beta-catenin signaling.

Re: the reviewer's comment that "The ChIP seq data referenced does not prove this to be the case." We agree. We think that our conclusion in the Results section is appropriately cautious: "Taken together, these in vivo analyses support a model in which Wnt7a and Tbx3 act in the same pathway. They are, therefore, consistent with the model of Zimmerli et al (2020) in which TBX3 participates directly in the transcriptional response to beta-catenin signaling in the developing limb."

#8 A further interesting observation is the patches of clusters of cells with different Lmx1b expression, but this is not expanded upon.

© 2022. Published by The Company of Biologists under the terms of the Creative Commons Attribution License (https://creativecommons.org/licenses/by/4.0/).

REPLY. We agree – this patchiness is fascinating. Our guess is that there is a thresholding process: cells with beta-catenin signaling that is above a certain threshold will express Lmx1b and cells below a certain threshold will not. One would need to additionally postulate that there is some form of communication between neighboring cells that mutually reinforces the high or low beta-catenin states so that the cells with or without Lmx1b expression tend to occur together spatially (i.e. in clusters).
The first part of this idea (thresholding) is exactly what we see in the CNS vasculature in response to reduced levels of beta- catenin signaling: endothelial cells are quantized with respect to expression (or not) of blood brain barrier markers (Wang, Y., Rattner, A., Zhou, Y., Williams, J., Smallwood, P.M., and Nathans, J. (2012) Norrin/Frizzled4 signaling in retinal vascular development and blood brain barrier plasticity. Cell 151: 1332-1344.). In the initial submission, we thought this might be too speculative to discuss, but we have now added these ideas to the Discussion.

#9 The justification for scoring one point for a missing digit and 2 points for a missing fibula/ulna is unclear.

REPLY. Thank you for pointing that out. We have added a sentence to the Results to expand on the scoring system. Missing digits are seen without a missing fibula/ulna, but a missing fibula/ulna is always accompanied by missing digits. Therefore, we consider a missing fibula/ulna to be evidence of a more severe defect, hence 2 points. All scoring systems have an element of arbitrariness, but this one strikes us as a good reflection of phenotypic severity, as judged by visual inspection.

Reviewer 2 Advance Summary and Potential Significance to Field:
Wang and colleagues dissect the contribution of WNT7A, WNT7B, RECK, and GPR124 in limb development throughcomprehensive gene dosage experiments. The authors thoroughly characterize combinations of hypomorphic, null, and conditional alleles of Wnt7a, Wnt7b, Reck, and Gpr124. Bone architecture, ectopic growth of nail-like structures, and marker gene expression are investigated in embryonic and adult mice. Overall, the data quality is very high (a strength of the study lies in its comprehensive nature). The paper is very well written, and the findings are interesting. The physiological role of this signaling axis has so far mainly been detailed in CNS vascular endothelial cells. Identifying a novel developmental setting controlled by RECK and GPR124 represents an important finding. We, therefore, recommend publication in Development, provided that the following comments are considered:

Reviewer 2 Comments for the Author:
Major comments: #1. In its present form, the paper does not provide direct evidence that RECK and GPR124 control limb development by activating beta-catenin signaling. Conditional restoration of a constitutively-active beta-catenin allele and/or the analysis of Wnt activity reporter mice or staining for established Wnt target genes should be attempted (e.g. Axin2, Lef1). This is particularly important as GPR124 and RECK seem to have different impacts on limb development. Direct analysis of Wnt activity might explain such differences.

REPLY. We agree that most of the data that we present is genetic and, therefore, we are inferring mechanism from genetic analyses of phenotypes. That said, we think that the limb phenotype caused by the CRISPR-generated PW allele of Reck and the Reck-Wnt7a genetic interactions are especially strong evidence for beta-catenin signaling as the mechanism. This is because the PW allele changes only two surface residues (proline256-to-alanine and tryptophan261-to-alanine) on the CC4 domain of Reck that specifically renders the protein incapable of Wnt7a/Wnt7b stimulation. The PW allele has no effect on the yield or cell surface localization of Reck in transfected cells, no effect on Reck levels by Western blotting, and presumably no effect on the structure or function of other Reck domains (e.g. the metalloproteinase inhibitor domain). The design and characterization of the PW allele is described in detail in Cho, C., Wang, Y., Smallwood, P.M., Williams, J., and Nathans, J. (2019) Molecular determinants in Frizzled, Reck, and Wnt7a for ligand-specific signaling in neurovascular development. eLife 8: e47300. We have now expanded the Results section to make this clear, including the sentence “Therefore, we infer that any phenotypes caused by the ReckPW allele arise from a decrease in WNT7A/WNT7B signaling.”.

The experiment that the reviewer suggests – bypassing a defect at the cell surface by using a constitutively active beta-catenin – is logical. Indeed, we have used this strategy extensively in studying beta-catenin signaling in vascular endothelial cells. However, in the vascular system, we

© 2022. Published by The Company of Biologists under the terms of the Creative Commons Attribution License (https://creativecommons.org/licenses/by/4.0/).

know which cell type to target for beta-catenin activation (vascular endothelial cells) and we have specific CreER lines to do that. By contrast, in the limb, the cells that are receiving the Wnt7a/Wnt7b signal are presumably a subset of mesenchymal cells. As a result, we do not know which CreER drivers to use or whether such drivers even exist. For the beta-catenin rescue experiment to work, it would need to be done precisely in space and time, since excess beta-catenin signaling has its own phenotypes, such as excessive cell proliferation. As a complicating factor for this type of experiment, there are multiple Wnts in addition to Wnt7a and Wnt7b that are expressed in the developing limb, and presumably there are also multiple classes of cells responding with beta-catenin stabilization. Artificially activating beta- catenin in those cells would likely generate additional phenotypes.

With respect to looking at Wnt/beta-catenin responsive genes, we have used immunostaining to analyze the protein encoded by Lmx1b, the most relevant Wnt7a/Wnt7b target gene, which is downstream of Wnt signaling in the dorsal limb and is responsible for D/V patterning. Our immunostaining data show that Reck mutation or Wnt7a mutation leads to similar reductions in Lmx1b expression in the distal limb and also to characteristic changes in its spatial distribution. We have expanded our earlier LMX1B immunostaining analysis at E13 (Figure 8) by adding a new Supplemental Figure 4 which shows LMX1B immunostaining in limb whole mounts at E11. The E11 data shows similar defects in LMX1B distribution caused by Reck mutation or Wnt7a mutation.

Regarding the analysis of other Wnt-responsive genes, our approach has been to analyze limb gene expression in a comprehensive and quantitative manner with single nucleus (sn)RNAseq. These analyses are still in progress, and we are planning to publish them as a separate study, which will include multiple time points and analyses of multiple mutants.
However, we can share some preliminary data with the editor and the reviewers (appended to this letter), which supports the conclusion that Reck and Wnt7a/Wnt7b loss of function mutations lead to the same molecular defects. The appended Figure shows snRNAseq UMAP plots from E13 forelimbs from WT, Wnt7a-/-;Wnt7b+/- (labeled “Wnt7”), and Reck ex2del/PW embryos (labeled “Reck”). The Wnt7a-/-;Wnt7b+/- and Reck ex2del/PW embryos have similarly severe limb phenotypes. For each mutant, the UMAP was generated from two independent replicates, with >17,000 nuclei sequenced per genotype. The data were normalized with Seurat SCTransform to minimize batch effects (see Hafemeister, C. & Satija, R. 2019.
Normalization and variance stabilization of single-cell RNA-seq data using regularized negative binomial regression. Genome Biol 20, 296). Panel (A) shows Ctnnb1 (beta-catenin) and Actb (actin), which serve as controls and which show indistinguishable levels and patterns of gene expression in the three genotypes. Panel (B) shows that Reck mutation has no effect on expression of Wnt7a or Wnt7b (red arrows point to the epithelial cell cluster, the site of Wnt7a and Wnt7b production). Interestingly, homozygosity for the KO allele of Wnt7a, which apparently does not reduce the levels of the internally deleted transcripts in epithelial cells, leads to a modest induction of Wnt7a transcript abundance in mesenchymal cells (the large central cluster). Panel (C) shows the effects of Wnt7a/Wnt7b and Reck mutation on Wnt-responsive genes. Lmx1b shows the largest changes in the lower left region of the mesenchymal cluster (blue arrows). Axin2, Apcdd1, Nkd1 (Naked1), and Lef1 show only modest reductions in transcript levels in the same region of the mesenchymal cluster in both Wnt7a-/-;Wnt7b+/-, and Reck ex2del/PW embryos.

Completing the snRNAseq analysis will require a lot more work, including many more limbs, earlier time points, additional genotypes, additional computational analyses, and various validation experiments. We would prefer to present the single cell analysis and related experiments as a separate and comprehensive study, and we hope that it will serve as a useful resource for the limb development community.

With respect to the difference between Reck and Gpr124 phenotypes, this is addressed in response to comment #5 from Reviewer #1.

#2. Reduced Reck has been shown to downregulate Wnt7a expression in the forelimb at E11.5 (Yamamoto et al. 2012). Based on microarray evidence, ~35% reduction in Reck expression triggers a similar ~35% reduction in Wnt7a mRNA levels. This reduction seems even more drastic when assessed by ISH. Some of the Reck phenotypes reported in this paper could thus be a consequence of reduced expression of the ligand rather than the co-receptor itself. For example, the data presented in Fig. 3 are difficult to interpret without a direct measure of Wnt7a and Wnt7b

© 2022. Published by The Company of Biologists under the terms of the Creative Commons Attribution License (https://creativecommons.org/licenses/by/4.0/).

expression level and distribution. This question should be addressed experimentally by probing the distribution of Wnt7a and Wnt7b mRNA in a selected set of genetic conditions (RT-PCR, ISH, RNAscope, immunostaining). Data interpretation should be adjusted accordingly.

REPLY. The reviewer is referring to Figures 5C and 5E of Yamamoto et al (2012) Biology Open 1: 458-466). We are not sure how to compare this data to ours. The main difference is that we studied a CRISPR-generated ReckPW allele that is specifically defective in beta-catenin signaling, whereas Yamamoto et al studied Reck compound heterozygotes for one allele that reduces Reck levels and a second allele that is null. Thus, Yamamoto at al were observing phenotypes that reflect a reduction in all of Reck's multiple domains and activities. With respect to the 35% reduction in Wnt7a mRNA level reported by Yamamoto et al (Figure 5E), we are wary of ascribing biological significance to such a modest reduction because Wnt7+/- mice (presumably with a 50% reduction in mRNA) exhibit no limb phenotype. As the reviewer notes, the in situ hybridization image in Yamamoto et al (Figure 5C) appears to show >35% reduction, which seems to be inconsistent with the micro-array data shown in Yamamoto et al Figure 5E. As noted in the answer to comment #1 from this reviewer (above) we have been approaching the gene expression analyses in what we think will be a more comprehensive and quantitative manner with snRNAseq. In particular, we think that snRNAseq analyses will be more powerful than total limb bud RT-PCR or ISH because it provides quantitative data for each cell cluster across the entire transcriptome. As shown in the accompanying UMAP figure, Wnt7a and Wnt7b expression is unaffected or minimally affected by Reck mutation.

#3. The data reveal that Wnt7a controls limb development at least partially independently of Gpr124, and maybe Reck. As a most striking illustration, constitutive null Gpr124 mutants only exhibit mild phenotypes, that are exacerbated by the loss of Wnt7a (Fig. 2). The situation of Reck is less clear as conditional null alleles are not available in this study. However, a similar trend is noted in homozygous Reck hypomorphic mutants, which only exhibit modest developmental limb defects, that are exacerbated by Wnt7a loss of function (Fig. 3). Other Reck alleles combinations reveal stronger phenotypes (Fig. 4). The non- essential role of Gpr124 (and perhaps Reck) contrasts somewhat with the situation prevailing in the CNS, where the endothelial-specific loss of Gpr124 or Reck, more closely mirrors the consequences of Wnt7a/b combined deletion. We believe that the Gpr124 (and possibly Reck)-independent Wnt7a/b signaling occurring in the limb should be emphasized by the authors, as it might have important consequences on the interpretation of some key experiments (see the previous comment).

REPLY. Thank you for that analysis. We mostly agree. We have now added a new paragraph to the Discussion to comment on it. This question was also raised in comment #5 by reviewer #1. Please see the reply to that comment.

4. The parametric statistical test used throughout this study (Student's t-test) is not suitable for analyzing the discrete (non- continuous) values of the embryonic limb phenotypic scoring system. Non-parametric analyses should be performed instead. This will likely affect some of the conclusions.

REPLY. Thank you for pointing out this error! We have recalculated all of the statistical analyses using the non-parametric Mann-Whitney-Wilcoxon test and updated the figures. While the conclusions are unchanged, the P-values are more modest with the non-parametric analysis.

Minor comments:
1. Last paragraph of the introduction. We suggest detailing what the hypomorphic Reck mutants are (nature of the deletion, functional consequence).

REPLY. Thank you. We have done that.

2. Page 4. Please correct 'embryos have a skeletal defects'. REPLY. Thank you. We have done that.

Reviewer 3 Advance Summary and Potential Significance to Field:
In this manuscript titled "The WnT7A/WNT7B/GPR124/RECK signaling module plays an essential role in mammalian limb development", Wang and colleagues explored the genetic interactions between the canonical Wnt ligands WNT7A and WNT7B and their cell surface co-activators RECK

© 2022. Published by The Company of Biologists under the terms of the Creative Commons Attribution License (https://creativecommons.org/licenses/by/4.0/).

and GPR124 in mouse limb development. This a tour de force study in which the authors used a large number of allelic combinations to demonstrate that WNT7A, WNT7B, RECK and GPR124 function in the same pathway to mediate canonical Wnt signaling and orchestrate proper limb development. This work complements previous studies showing that these molecules work in concert to media canonical Wnt signaling during angiogenesis, and further supports that the function of the WNT7/RECK/GPR124 signaling module is conserved across multiple tissue/organ systems. In addition, the study provides new insights on the broader questions of how signaling specificity is achieved within the Wnt signaling system, and how a relatively small number of developmental pathways can be reused and repurposed to drive diverse developmental processes. Overall, the quality of the study is excellent both in terms of its conceptual advancement and its experimental rigor.

Reviewer 3 Comments for the Author:
Addressing the comments below would improve the quality of the manuscript:

1. Can the authors mention how many animals were analyzed for the deformities as well as what the bars represent in the limb phenotype scoring analyses shown in Figs 1-4 (SE or SD)?

REPLY. All of the bars represent standard deviation (SD). We have added that to the revised figure legends. We have prepared a supplemental Table 1 that lists the number of animals (embryos) of each genotype analyzed in Figures 1-4 and 6. For some genotypes, there is considerable overlap of the many data points in the plots. In the revised figures, we have manually shifted some of the data points laterally so that the reader can see them more clearly, but many still overlap.

2. Some of the p values, particularly in Figure 4 seem unusually high. For example, the p value for Gpr124+/+; ReckΔ/Δ (blue) and Gpr124+/+; ReckΔ/PW (salmon) is mentioned as 2.7x10^-11, though the difference does not seem to approach that degree of significance. On the other hand, the comparison between Gpr124+/-; Reck+/+ (light green) and Gpr124+/-; ReckΔ/Δ (dark green), which seems to be highly significant, has a p value of 6.4x10^-4. Can the authors provide clarifications on this?

REPLY. As pointed out by reviewer 2, we erroneously used a parametric test (the student t-test) when we should have used a non-parametric test (e.g. the Mann-Whitney-Wilcoxon test) for the P-value calculation. We have recalculated all of the statistical analyses using the Mann-Whitney-Wilcoxon test and updated the figures. While the conclusions are unchanged, the P-values are more modest with the non-parametric analysis. Regarding the reviewer's specific examples: (1) the non-parametric P-value for Gpr124+/+; ReckΔ/Δ (blue) vs. Gpr124+/+; ReckΔ/PW (salmon) is $6.6x10^{-8}$. This P-value is low because there are so many data points (embryos): n=25 embryos for each the two genotypes. (2) The non-parametric P- value for Gpr124+/-; Reck+/+ (light green) vs. Gpr124+/-; ReckΔ/Δ (dark green) is now 5.3×10^{-6}. The number of data points (embryos) is 16 and 7, respectively. The P-value is so low because the phenotype score for all 16 of the Gpr124+/-; Reck+/+ embryos is zero. We have added a Supplemental Table 1 to show the number of embryos in Figures 1-4 and 6, i.e. all of the embryo cohorts that were used for P-value calculations.

Thank you for overseeing the review of this manuscript.

NOTE: We have removed unpublished data that had been provided for the referees in confidence.

© 2022. Published by The Company of Biologists under the terms of the Creative Commons Attribution License (https://creativecommons.org/licenses/by/4.0/).

Second decision letter

MS ID#: DEVELOP/2021/200340

MS TITLE: The WNT7A/WNT7B/GPR124/RECK Signaling Module Plays an Essential Role in Mammalian Limb Development

AUTHORS: Jeremy Nathans, Yanshu Wang, Arjun Venkatesh, Jiajia Xu, Mingxin Xu, John Williams, Philip M. Smallwood, and Aaron James

I have now received all the referees reports on the above manuscript, and have reached a decision. The referees' comments are appended below, or you can access them online: please go to BenchPress and click on the 'Manuscripts with Decisions' queue in the Author Area.

The overall evaluation is very positive and we would like to publish a revised manuscript in Development. However as you will see Reviewer 1 feels that there are some issues raised during the first review that have not been addressed in the revision. I'm returning the manuscript to you to give you the opportunity to further modify your manuscript should you feel it appropriate. If you do not agree with any of their criticisms or suggestions explain clearly why this is so. Your manuscript will not require any further review, rather I will look it over myself prior to acceptance.

Reviewer 1

Advance summary and potential significance to field

see original response

Comments for the author

Two key considerations in examining and interpreting the compound, conditional deletions of Wnt7a and 7b are (i) their respective expression temporal and spatial expression patterns in the limb, when and where their expression may overlap or be unique in space and time and (ii) the dynamics of the cre delete transgenes employed to disrupt gene expression.
No details are provided or discussed on the potential areas of gene expression overlap and therefore possible functional overlap and how this could be distinguished using the ectodermally-restricted cre driver (Msx)(and the more broadly expressed Cdx2 cre). It is also worth noting that the Msx2Cre would not be expressed throughout the limb ectoderm. This was raised in our original review and by other reviewers, but unfortunately has not been addressed to our satisfaction.
Overall, the revisions to the manuscript include some relatively minor text modifications and we feel many of the key issues that were raised in our original review have remained unchanged within the manuscript. The data and how it is represented and interpreted remains unchanged from the original version.
The almost exclusive analysis of relatively late stage skeletal phenotypes using alcian blue staining and absence of any analysis of changes in expression of gene molecular markers of limb patterning means interpretation of the results is limited.
“Bleeding into the digits” remains an unsatisfactory description of a phenotype. It remains unclear what this actually is and what is causing it.

Reviewer 2

Advance summary and potential significance to field

Wang and colleagues dissect the contribution of WNT7A, WNT7B, RECK, and GPR124 in limb development through comprehensive gene dosage experiments. The authors thoroughly characterize combinations of hypomorphic, null, and conditional alleles of Wnt7a, Wnt7b, Reck, and Gpr124. Bone architecture, ectopic growth of nail-like structures, and marker gene expression are investigated in embryonic and adult mice. Overall, the data quality is very high (a strength of the study lies in its comprehensive nature). The paper is very well written, and the findings are

© 2022. Published by The Company of Biologists under the terms of the Creative Commons Attribution License (https://creativecommons.org/licenses/by/4.0/).

interesting. The physiological role of this signaling axis has so far mainly been detailed in CNS vascular endothelial cells. Identifying a novel developmental setting controlled by RECK and GPR124 represents an important finding.

Comments for the author

The authors addressed my concerns, and the revised manuscript is now a strong candidate for Development.

<u>Reviewer 3</u>

Advance summary and potential significance to field

In this manuscript titled “The WnT7A/WNT7B/GPR124/RECK signaling module plays an essential role in mammalian limb development”, Wang and colleagues explored the genetic interactions between the canonical Wnt ligands WNT7A and WNT7B and their cell surface co-activators RECK and GPR124 in mouse limb development. This a tour de force study in which the authors used a large number of allelic combinations to demonstrate that WNT7A, WNT7B, RECK and GPR124 function in the same pathway to mediate canonical Wnt signaling and orchestrate proper limb development. This work complements previous studies showing that these molecules work in concert to media canonical Wnt signaling during angiogenesis, and further supports that the function of the WNT7/RECK/GPR124 signaling module is conserved across multiple tissue/organ systems. In addition, the study provides new insights on the broader questions of how signaling specificity is achieved within the Wnt signaling system, and how a relatively small number of developmental pathways can be reused and repurposed to drive diverse developmental processes. Overall, the quality of the study is excellent both in terms of its conceptual advancement and its experimental rigor.

Comments for the author

This is revision of a manuscript that I previously reviewed and commented. I was enthusiastic about the study and made suggestions for further improvements. The authors have now appropriately addressed all my suggestions/comments, and the manuscript is ready for publication in my opinion. Again this is an elegant study with interesting and novel findings, and the manuscript is very well written.

Second revision

<u>Author response to reviewers' comments</u>

We are grateful to you and the reviewers for your thoughtful and constructive suggestions to improve the manuscript.

In your letter of April 14, 2022, you wrote: “The overall evaluation is very positive and we would like to publish a revised manuscript in Development. However as you will see Reviewer 1 feels that there are some issues raised during the first review that have not been addressed in the revision. I'm returning the manuscript to you to give you the opportunity to further modify your manuscript should you feel it appropriate. If you do not agree with any of their criticisms or suggestions explain clearly why this is so. Your manuscript will not require any further review, rather I will look it over myself prior to acceptance.”

Reviewers 2 and 3 had no further suggestions, but Reviewer 1 had four comments to which we have responded below. In response to these comments, we have made some additions to the text at the start of the Results section. These are shown in red in the revised text document.

© 2022. Published by The Company of Biologists under the terms of the Creative Commons Attribution License (https://creativecommons.org/licenses/by/4.0/).

Reviewer 1 Comments for the author

(1) Two key considerations in examining and interpreting the compound, conditional deletions of Wnt7a and 7b are (i) their respective expression temporal and spatial expression patterns in the limb, when and where their expression may overlap or be unique in space and time and (ii) the dynamics of the cre delete transgenes employed to disrupt gene expression.
No details are provided or discussed on the potential areas of gene expression overlap and therefore possible functional overlap and how this could be distinguished using the ectodermally-restricted cre driver (Msx)(and the more broadly expressed Cdx2 cre). It is also worth noting that the Msx2Cre would not be expressed throughout the limb ectoderm. This was raised in our original review and by other reviewers, but unfortunately has not been addressed to our satisfaction.
(2) Overall, the revisions to the manuscript include some relatively minor text modifications and we feel many of the key issues that were raised in our original review have remained unchanged within the manuscript. The data and how it is represented and interpreted remains unchanged from the original version.

(3) The almost exclusive analysis of relatively late stage skeletal phenotypes using alcian blue staining and absence of any analysis of changes in expression of gene molecular markers of limb patterning means interpretation of the results is limited.

(4) “Bleeding into the digits” remains an unsatisfactory description of a phenotype. It remains unclear what this actually is and what is causing it.

REPLY. We divide our reply into four parts (labeled 1-4) to address each of the four sections of the reviewer’s comments above.

(1) We thank the reviewers for pointing this out. They are right: there is more to say about the Wnt7b and Msx2-Cre expression patterns, and we have revised the first part of the Results section, which is related to Figure 1, accordingly (see below).

We note that the expression patterns of Wnt7a, Wnt7b, Cdx2-Cre, and Msx2-Cre have been published, and we have cited the relevant papers in our manuscript. These are listed below:

Wnt7a and Wnt7b expression:

Parr BA, McMahon AP. 1995. Dorsalizing signal Wnt-7a required for normal polarity of D-V and A-P axes of mouse limb. Nature 374:350-353.

Summerhurst K, Stark M, Sharpe J, Davidson D, Murphy P. 2008. 3D representation of Wnt and Frizzled gene expression patterns in the mouse embryo at embryonic day 11.5 (Ts19). Gene Expr Patterns 8:331-348.

Witte F, Dokas J, Neuendorf F, Mundlos S, Stricker S. 2009. Comprehensive expression analysis of all Wnt genes and their major secreted antagonists during mouse limb development and cartilage differentiation. Gene Expr Patterns 9:215-23.

Cdx2-Cre expression:

Chang H, Smallwood PM, Williams J, Nathans J. 2016. The spatio-temporal domains of Frizzled6 action in planar polarity control of hair follicle orientation. Dev Biol 409:181-193.

Hinoi T, Akyol A, Theisen BK, Ferguson DO, Greenson JK, Williams BO, Cho KR, Fearon ER. 2007. Mouse model of colonic adenoma-carcinoma progression based on somatic Apc inactivation. Cancer Res 67:9721–9730.

Suh J, Eom JH, Kim NK, Woo KM, Baek JH, Ryoo HM, Lee SJ, Lee YS. 2019. Growth differentiation factor 11 locally controls anterior-posterior patterning of the axial skeleton. J Cell Physiol 234:23360-23368.

Msx2-Cre expression:

© 2022. Published by The Company of Biologists under the terms of the Creative Commons Attribution License (https://creativecommons.org/licenses/by/4.0/).

Sun X, Lewandoski M, Meyers EN, Liu YH, Maxson RE Jr, Martin GR. 2000. Conditional inactivation of Fgf4 reveals complexity of signalling during limb bud development. Nat Genet 25:83-86.

In the original description of Msx2-Cre by Sun et al (2000), the authors used a Z/AP Cre-reporter to characterize Cre expression and write: "In prospective forelimb, we detected AP activity in a few ectoderm cells at the 24-somite stage, and throughout the nascent AER by the 29-somite stage. In prospective hindlimb, AP activity was widespread in the ectoderm beginning at the 24- somite stage (Fig. 1e,f). By the 38-somite stage, we detected high AP activity throughout forelimb and hindlimb AERs and ventral ectoderm (Fig. 1g)."

Witte et al (2009) analyzed Wnt7b expression by in situ hybridization and write: "Wnt7b, in contrast to its well-described close relative Wnt7a, shows an expression in both dorsal and ventral ectoderm at early stages E10.5–E11.5, however, we noted that at E11.5 expression of Wnt7b was weaker in dorsal than in ventral ectoderm (Fig. 1P and Supplementary Fig. 4, arrow). This is in accordance with the finding of Summerhurst et al. (2008) of predominant ventral expression at E11.5. In later stages, Wnt7b becomes restricted to basal cells of the epidermis (Fig. 1Qand R) and shows weak expression in the perichondrium flanking the prehypertrophic chondrocytes (Supplementary Fig. 4, arrowhead) as described before (Hu et al., 2005)."

We have now briefly summarized these patterns of expression in the Results section. Reviewer 1 wrote about "...the potential areas of gene expression overlap and therefore possible functional overlap and how this could be distinguished using the ectodermally-restricted cre driver (Msx) and the more broadly expressed (Cdx2 cre)". Since these two Cre drivers produced the same phenotypic severity when combined with Wnt7a null and Wnt7b CKO alleles (quantified in Figure 1C), the conclusion is fairly simple: the cells that serve as the principal source of WNT7B are located in that part of the ectoderm where Msx2-Cre is active. Cdx2-Cre, which serves as a positive control, recombines essentially all cells within the developing hindlimb. To make this conclusion crystal clear, we have added the following sentence to the last paragraph of the Results section describing Figure 1: "The close similarity in phenotypic severity produced by Cdx2-Cre and Msx2-Cre drivers indicates that the principal source of WNT7B is the ectodermal territory where Msx2-Cre is active."

(2) Reviewer #1 wrote: "The data and how it is represented and interpreted remains unchanged from the original version." We beg to differ. We made numerous text and figure changes and additions in response to the reviewers' comments. To cite one example of our response to the first round of reviewer critiques related to data interpretation, we added new text to the Results section and a new paragraph to the Discussion section to describe and discuss the meaning of the more modest limb phenotype seen with loss of Gpr124 compared to loss of Reck.

(3) While this comment is correct, it somewhat misses the point. It is true that we have analyzed tissue patterning and cell fate phenotypes that reside downstream of ligands/receptors/transcription factors/chromatin structure, etc, but that is a perfectly legitimate approach when one is conducting a genetic analysis. Importantly, we have been careful not to overstate the data or the conclusions.

(4) "Bleeding into the digits". We disagree with the reviewer's assessment. This is a minor facet of the phenotype, but it is a real phenomenon and we have described it as we see it. It is an intriguing part of the picture because it suggests that there might be a mechanistic link between WNT7A/WNT7B signaling in vascular development in the CNS (which also shows a bleeding phenotype) and vascular development in the limbs.

Thank you for overseeing the review of this manuscript.

© 2022. Published by The Company of Biologists under the terms of the Creative Commons Attribution License (https://creativecommons.org/licenses/by/4.0/).

Third decision letter

MS ID#: DEVELOP/2021/200340

MS TITLE: The WNT7A/WNT7B/GPR124/RECK Signaling Module Plays an Essential Role in Mammalian Limb Development

AUTHORS: Jeremy Nathans, Yanshu Wang, Arjun Venkatesh, Jiajia Xu, Mingxin Xu, John Williams, Philip M. Smallwood, and Aaron James
ARTICLE TYPE: Research Article

I am happy to tell you that your manuscript has been accepted for publication in Development, pending our standard ethics checks.

© 2022. Published by The Company of Biologists under the terms of the Creative Commons Attribution License (https://creativecommons.org/licenses/by/4.0/).
